# Loss of androgen signaling in mesenchymal sonic hedgehog responsive cells diminishes prostate development, growth, and regeneration

**DOI:** 10.1371/journal.pgen.1008588

**Published:** 2020-01-13

**Authors:** Vien Le, Yongfeng He, Joseph Aldahl, Erika Hooker, Eun-Jeong Yu, Adam Olson, Won Kyung Kim, Dong-Hoon Lee, Monica Wong, Ruoyu Sheng, Jiaqi Mi, Joseph Geradts, Gerald R. Cunha, Zijie Sun

**Affiliations:** 1 Department of Cancer Biology, Beckman Research Institute of City of Hope, Duarte, California, United States of America; 2 Department of Population Sciences, Beckman Research Institute of City of Hope, Duarte, California, United States of America; 3 Department of Urology, School of Medicine, University of California San Francisco, San Francisco, California, United States of America; Stanford University School of Medicine, UNITED STATES

## Abstract

Prostate embryonic development, pubertal and adult growth, maintenance, and regeneration are regulated through androgen signaling-mediated mesenchymal-epithelial interactions. Specifically, the essential role of mesenchymal androgen signaling in the development of prostate epithelium has been observed for over 30 years. However, the identity of the mesenchymal cells responsible for this paracrine regulation and related mechanisms are still unknown. Here, we provide the first demonstration of an indispensable role of the androgen receptor (AR) in sonic hedgehog (SHH) responsive Gli1-expressing cells, in regulating prostate development, growth, and regeneration. Selective deletion of AR expression in Gli1-expressing cells during embryogenesis disrupts prostatic budding and impairs prostate development and formation. Tissue recombination assays showed that urogenital mesenchyme (UGM) containing AR-deficient mesenchymal Gli1-expressing cells combined with wildtype urogenital epithelium (UGE) failed to develop normal prostate tissue in the presence of androgens, revealing the decisive role of AR in mesenchymal SHH responsive cells in prostate development. Prepubescent deletion of AR expression in Gli1-expressing cells resulted in severe impairment of androgen-induced prostate growth and regeneration. RNA-sequencing analysis showed significant alterations in signaling pathways related to prostate development, stem cells, and organ morphogenesis in AR-deficient Gli1-expressing cells. Among these altered pathways, the transforming growth factor β1 (TGFβ1) pathway was up-regulated in AR-deficient Gli1-expressing cells. We further demonstrated the activation of TGFβ1 signaling in AR-deleted prostatic Gli1-expressing cells, which inhibits prostate epithelium growth through paracrine regulation. These data demonstrate a novel role of the AR in the Gli1-expressing cellular niche for regulating prostatic cell fate, morphogenesis, and renewal, and elucidate the mechanism by which mesenchymal androgen-signaling through SHH-responsive cells elicits the growth and regeneration of prostate epithelium.

## Author summary

Prostate formation, growth, and regeneration, as well as tumorigenesis, depend on androgens and androgen receptor (AR)-mediated signaling pathways. Tissue recombination assays done more than 30 years ago demonstrated a decisive role for stromal androgen signaling in prostatic epithelium development. However, in the intervening time, the identity of the mesenchymal cells in the urogenital sinus mesenchyme that convey androgen signaling and control prostate epithelium development, morphogenesis, and regeneration has not been determined. In this study, using mouse genetic tools, we demonstrate for the first time that selective deletion of AR in mesenchymal Gli1-expressing cells abolishes early development of prostate tissue and normal prostate formation, and diminishes prostate pubertal growth and regeneration. In addition, using tissue recombination assays, we directly determined an essential requirement for AR expression in mesenchymal Gli1-expressing cells during prostate epithelium development. Our results not only resolve a 30-year-old scientific puzzle by identifying the mesenchymal cell properties of androgen-responsive cells that elicit development of the embryonic prostate epithelium, but also explore a new regulatory mechanism for androgen and Shh signaling-mediated cellular niches in regulating prostatic cell fate, growth, and renewal through paracrine regulation. Given the importance of sex hormone and hedgehog signaling pathways in human development and tumorigenesis, this study extends beyond the field of prostate biology, raising new questions underlying sex hormone and SHH signaling in development and tumorigenesis.

## Introduction

Prostate formation, growth, and regeneration depend on androgen receptor (AR)-mediated signaling pathways [1]. During embryogenesis, the AR is initially expressed in the urogenital sinus mesenchyme (UGM), and its expression subsequently extends to both the UGM and urogenital sinus epithelium (UGE) [1,2]. Early tissue recombination studies that were done over 30 years ago showed that AR-deficient UGM combined with wild type UGE failed to develop into a prostate [3–5], implicating a decisive role of mesenchymal AR signaling in inducing development of the prostatic epithelium through paracrine regulation. Androgen signaling still remains essential in the postnatal prostate, controlling maturation, growth, and regeneration through mesenchymal-epithelial interactions [6]. However, the precise mechanisms for the regulation are still unclear. Specifically, the identity of prostatic cells that convey androgen signaling and control prostate development, morphogenesis, and regeneration has remained unclear in past decades [7–9].

Emerging evidence has shown that sonic hedgehog (SHH) signaling plays a critical role in prostate development, homeostasis, and regeneration through mesenchymal–epithelial interactions [10,11]. The SHH growth factors and its downstream effectors are expressed in either murine prostatic epithelial or mesenchymal cells, respectively, during embryogenesis and adulthood [12,13]. However, a previous study has shown Gli1 expression is dispensable for prostate development [12,13]. A critical role of SHH signaling in regulating adult stem cells has been explored in many self-renewing organs, including the prostate [13–15]. The regulation through paracrine interactions between stem cells and their surrounding niche has also been observed in prostate development, growth, and regeneration [16,17]. However, the precise mechanisms for SHH signaling in regulating prostatic stem/progenitor cells through a paracrine mechanism still remain unclear. Specifically, the biological role of SHH signaling pathways in androgen-regulated prostate development, morphogenesis and regeneration has not been fully explored.

In this study, we demonstrate an indispensable role of androgen signaling in mesenchymal Gli1-expressing cells in regulating prostate early development, morphogenesis, and regeneration. Specifically, tissue recombination assays showed that urogenital mesenchyme (UGM) containing AR-deficient mesenchymal Gli1-expressing cells combined with wild type urogenital epithelium (UGE) failed to develop normal prostate tissue in the presence of androgens, revealing the decisive role of AR in mesenchymal SHH responsive cells in prostate development. Prepubescent deletion of AR expression in Gli1-expressing cells resulted in severe impairment of androgen-induced prostate growth and regeneration. Using RNA-sequencing approaches, we examined the changes of transcriptome profiles in AR-deficient Gli1-expressing cells, and identified significant alterations in signaling pathways related to prostate development, stem cells, and organ morphogenesis. The data presented in this study elucidate an innovative role of the AR in the Gli1-expressing cellular niche for regulating prostatic cell fate, growth, and renewal, and elucidate a new paracrine regulatory mechanism by which mesenchymal androgen-signaling elicits the growth of prostate epithelium.

## Results

### Deletion of AR expression in mesenchymal Gli1-expressing cells impairs prostatic budding during embryogenesis

**Fig 1:**
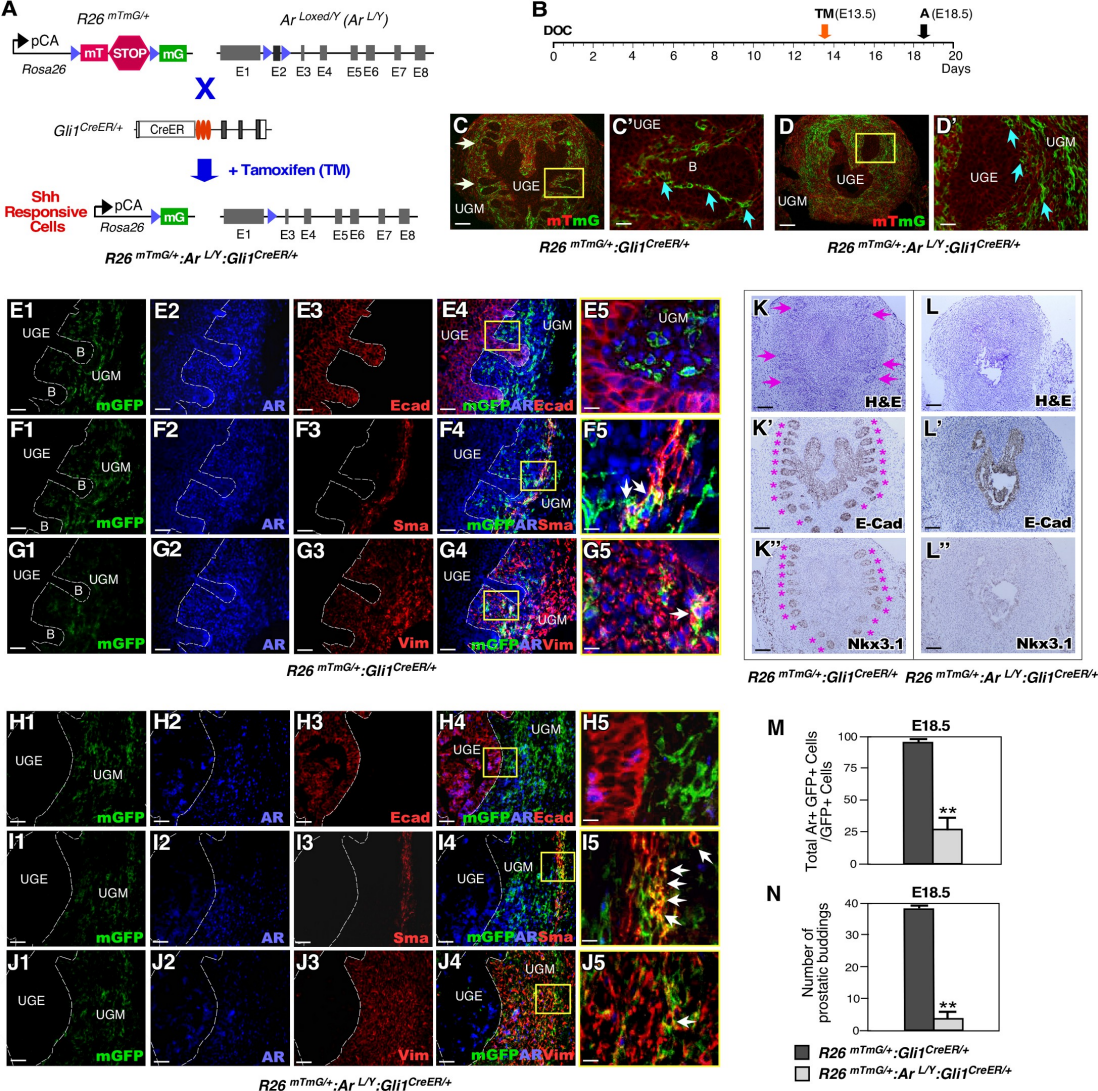
Deletion of AR in Gli1-expressing cells during embryogenesis disrupts prostatic bud formation. *(****A****)* Schematic for generating *Ar*^*L/Y*^:*Gli1*^*CreER/+*^, *R26*^*mTmG/+*^:*Gli1*^*CreER/+*^, and *R26*^*mTmG/+*^:*Ar*^*L/Y*^:*Gli1*^*CreER/+*^ mice. *(****B****)* Schematic of experimental timeline for TM induction and analysis. *(****C-D****)* Fluorescence micrographs of membrane-bound tdTomato (mT) and membrane-bound green fluorescent protein (mG) expression. White arrows indicate prostatic epithelial buds. Blue arrows indicate Gli1-derived GFP-positive cells adjacent to the UGE. UGE: urogenital sinus epithelium; UGM: urogenital sinus mesenchyme. Scale bars, C-D, 100 μm; C’-D’, 25 μm. *(****E1-J5****)* Triple-immunofluorescent images of UGS tissues isolated at E18.5 from indicated male embryos. White dashed lines indicate boundary between UGM and UGE, as well as epithelial buds “B”. White arrows indicate GFP mesenchymal positive cells. Scale bars, E1-4, F1-4, G1-4, H1-4, I1-4, and J1-4, 50 μm: E5, F5, G5, H5, I5, and J5, 12.5 μm. *(****K-K”****)* Either hematoxylin-eosin (H&E) and immunohistochemical (IHC) analyses of E18.5 UGS tissues of *R26*^*mTmG/+*^:*Gli1*^*CreER/+*^ embryos. Antibodies used for IHC are indicated in the lower right corner of images (please also see S6 Table). Pink arrows and asterisks (*) indicate normal prostatic epithelial buds. Scale bars, 100 μm. *(****L-L”****)* Similar analyses were performed as in K-K” with *R26*^*mTmG/+*^:*Ar*^*L/Y*^:*Gli1*^*CreER/+*^ UGS tissues. Scale bars, 100 μm. *(****M****)* Quantification of AR and mGFP double positive cells per mGFP-positive cells in indicated mouse UGS tissues, n = 4 embryos per genotype. Error bars indicate standard deviation (s.d.). **, *P* <0.01 (please also see supporting information provided in S1 Table). *(****N****)* Numbers of prostatic buds associated with each image of mouse UGS tissues of the indicated genotypes, n = 4 embryos per genotype. Detailed information for the quantification is included (S1 Table); **, *P* < 0.01; analyzed using 2-tailed *t*-test.

Expression of the AR first appears in the UGM before prostate morphogenesis and then extends to the UGE after budding and branching morphogenesis has begun [1,2]. Although the essential role of mesenchymal androgen signaling in prostate early development has been implicated for more than three decades [3,18], the identity of the cell types that are responsive to mesenchymal androgen signaling in UGM are still unknown. Given the critical role of the SHH signaling pathway in controlling prostate development [10,11], we evaluated if AR has a specific role in Shh-responsive Gli1-expressing cells during prostate early development. We developed *ROSA26R*^*mTmG/+*^:*Ar*^*LoxP/Y*^:*Gli1*^*CreERT2/+*^ (*R26*^*mTmG/+*^:*Ar*^*L/Y*^:*Gli1*^*CreER/+*^), and *Ar*^*LoxP/Y*^:*Gli1*^*CreERT2/+*^ (*Ar*^*L/Y*^:*Gli1*^*CreER/+*^) mice, as well as *R26*^*mTmG/+*^:*Gli1*^*CreER/+*^controls (Fig 1A). Upon tamoxifen (TM) injection, deletion of the *Ar* gene and activation of membrane-bound green fluorescent protein (mGFP) expression occur in Gli1-expressing cells in these mice (Fig 1A). This enabled us to trace the fate and pathological phenotype of Gli1-expressing cells. Because AR expression starts in the UGM at embryonic day 14 (E14), we administered TM at day E13.5 to pregnant females bearing embryos of the desired genotype and analyzed male UGS tissues at E18.5 (Fig 1B). mGFP expression was seen exclusively in UGM (Fig 1C and 1D), implicating the mesenchymal cellular properties of Gli1-expressing cells. Triple immunofluorescence (IF) analyses further showed Gli1-CreER-activated mGFP-expressing cells were exclusively located within the UGM (Fig 1E1-1J5), and that AR was co-expressed with the mesenchymal markers smooth muscle actin and vimentin in a subpopulation of mesenchymal AR- and Gli1-positive cells (arrows, Fig 1F4-5 and 1G4-5). In contrast to *R26*^*mTmG/+*^:*Gli1*^*CreER/+*^ controls, no prostatic budding was seen in UGS tissues from *R26*^*mTmG/+*^:*Ar*^*L/Y*^:*Gli1*^*CreER/+*^ and *Ar*^*L/Y*^:*Gli1*^*CreER/+*^ embryos (Fig 1D versus 1C, 1L versus 1K, and 1N). Immunohistochemical (IHC) analysis of the epithelial markers E-cadherin and Nkx3.1 further demonstrated the lack of prostatic epithelial buds in UGS tissues with AR-deficient Gli1-expressing cells (Fig 1K’–1K” and 1L’–1L”). Specific reduction of AR expression was also seen in mesenchymal Gli1-expressing cells of the above AR-deficient UGS tissues (arrows, Fig 1I4-5, 1J4-5 and 1M). These results provide a direct link between selective deletion of AR expression in mesenchymal Gli1-expressing cells and disruption of prostatic epithelium early development. Of note, we did not observe any phenotypic differences between the designed AR-mutant mice, *Ar*^*L/Y*^:*Gli1*^*CreER/+*^ and *R26*^*mTmG/+*^:*Ar*^*L/Y*^:*Gli1*^*CreER/+*^. Therefore, in all subsequent experiments we present representative data from *R26*^*mTmG/+*^:*Ar*^*L/Y*^:*Gli1*^*CreER/+*^ and *R26*^*mTmG/+*^:*Gli1*^*CreER/+*^mice.

### AR expression in mesenchymal Gli1-expressing cells is essential for androgen-mediated prostatic gland formation

**Fig 2:**
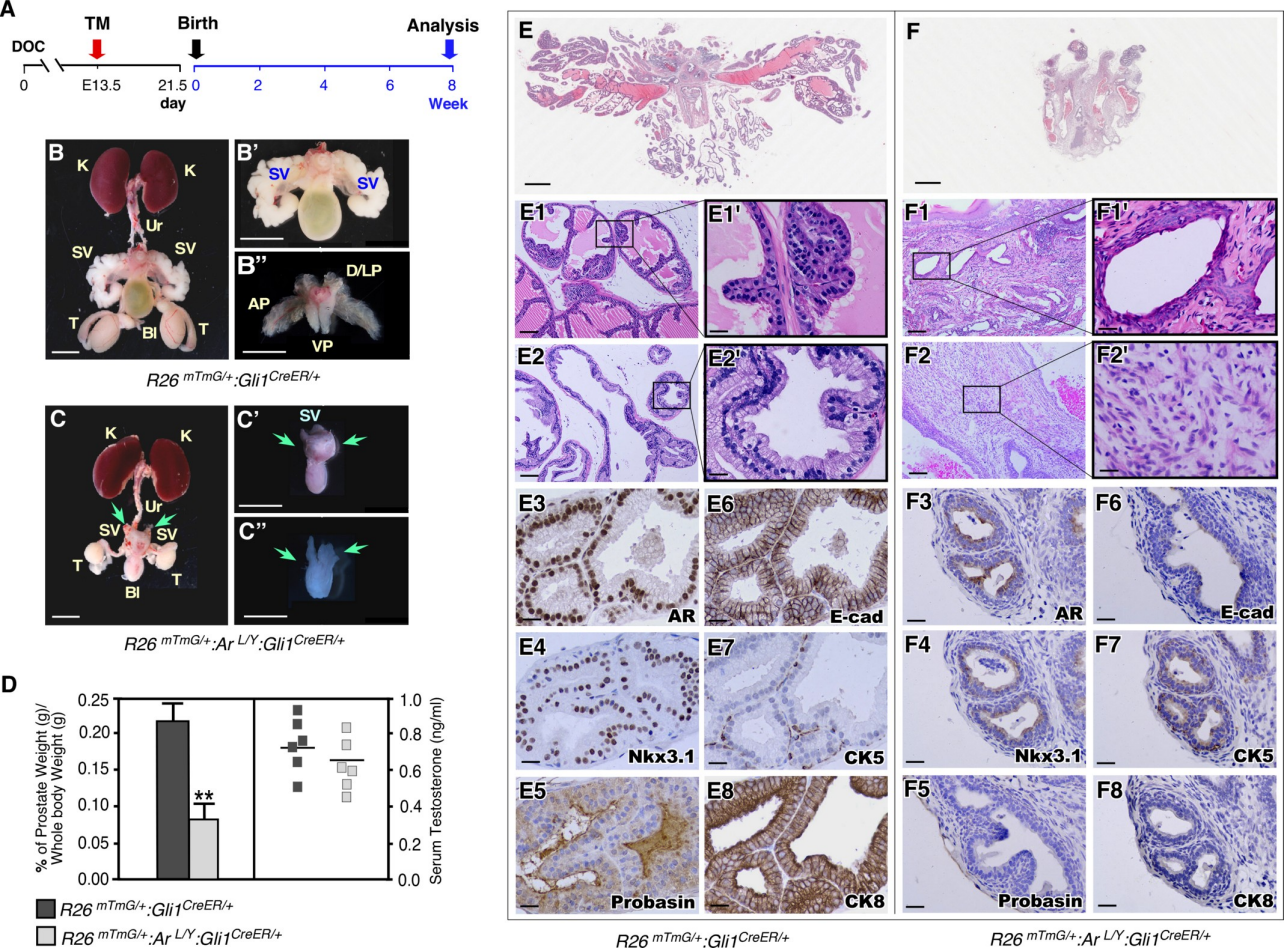
Deletion of AR in mesenchymal Gli1-expressing cells during embryogenesis diminishes prostate formation. ***(A)*** Experimental timeline, including conditional deletion of AR in Gli1-expressing cells during embryogenesis followed by postnatal analysis. ***(B*,*C)*** Representative images of urogenital tracts isolated from *R26*^*mTmG/+*^:*Gli1*^*CreER/+*^ and *R26*^*mTmG/+*^:*Ar*^*L/Y*^:*Gli1*^*CreER/+*^. Green arrows indicate abnormal and undeveloped seminal vesicle and prostatic lobes, C-C”. Scale bars, B-C, 1 cm; B’,B”,C’ and C”, 5 mm. K: kidney; Ur: ureter; SV: seminal vesicle; Bl: bladder; T: testicle; AP: anterior prostate; D/LP: dorsolateral prostate; VP: ventral prostate. ***(D)*** Ratio of prostate wet weight versus whole body weight as percentages (left) and serum testosterone concentrations (right) of mice of the indicated genotypes. Error bars indicate s.d.; **P < 0.01; analyzed using 2-tailed *t*-test; n = 6 mice per genotype. ***(E*,*F)*** Whole slide scan of H&E staining of prostate tissues from *R26*^*mTmG/+*^:*Ar*^*L/Y*^:*Gli1*^*CreER/+*^ and *R26*^*mTmG/+*^:*Gli1*^*CreER/+*^ age-matched control littermates. Scale bars, 1 mm. ***(E1*,*E2)*** Representative images of prostatic lobes from *R26*^*mTmG/+*^:*Gli1*^*CreER/+*^ mice. ***(F1*,*F2)*** Representative images of glandular elements which displayed squamous metaplasia and hypercellular stromal area from *R26*^*mTmG/+*^:*Ar*^*L/Y*^:*Gli1*^*CreER/+*^ mice. Scale bars, E1-F2, 100 μm; E1’-F2’, 20 μm. ***(E3-F8)*** Immunohistochemistry staining of prostatic tissues isolated from the indicated genotypes of mice with a number of prostate epithelial markers as labeled in images. Scale bars, 20 μm.

To evaluate the consequence of specific AR-deletion in mesenchymal Gli1-expressing cells during prostate formation, we administered TM to pregnant female mice at E13.5. Both AR-deficient and control newborns were fostered by wild-type females and analyzed at eight weeks of age (Fig 2A). Strikingly, the mature *R26*^*mTmG/+*^:*Ar*^*L/Y*^:*Gli1*^*CreER/+*^ male mice lacked any developed prostate lobes, while their control littermates showed normal prostate lobes (Fig 2C–2C” versus 2B–2B”). Prostate weights of the AR-deficient mice were significantly less than the controls (left panel, Fig 2D). The mutant mice also showed smaller testicles and abnormal seminal vesicles (Fig 2C versus 2B). However, serum testosterone levels showed no significant difference between the mutant and control mice (right panel, Fig 2D). Whole mount sections of mutant prostate glands showed a markedly simplified organ structure (Fig 2F versus 2E). Histologically, no typical prostate lobes and rare glandular elements were present in AR-deficient prostate tissues (Fig 2F1-2’), in contrast to those in age-matched male control littermates (Fig 2E1-2’). In addition to a diminished glandular compartment, the stroma in AR-deficient prostate tissues was more abundant and hypercellular, displaying patchy inflammatory infiltrate (Fig 2F2-2’) and squamous metaplasia (Fig 2F1-1’ and S1A and S1B Fig). IHC analyses showed very weak or no staining for prostate epithelial markers, including AR, probasin, Nkx3.1, E-cadherin, CK8 and CK5 in the urothelial tissues of AR deficient mice (Fig 2F3-8). In contrast, prostate tissues of control mice showed positive staining for the same markers (Fig 2E3-8). The complete lack of prostate structure in *R26*^*mTmG/+*^:*Ar*^*L/Y*^:*Gli1*^*CreER/+*^ mice demonstrates a severe prostatic defect that has never been observed in previous targeted AR-deficient murine models [7], and the indispensable role of mesenchymal AR in Gli1-expressing cells during early prostate development and formation.

### AR expression in mesenchymal Gli1-expressing cells is essential for prostatic epithelial development

**Fig 3:**
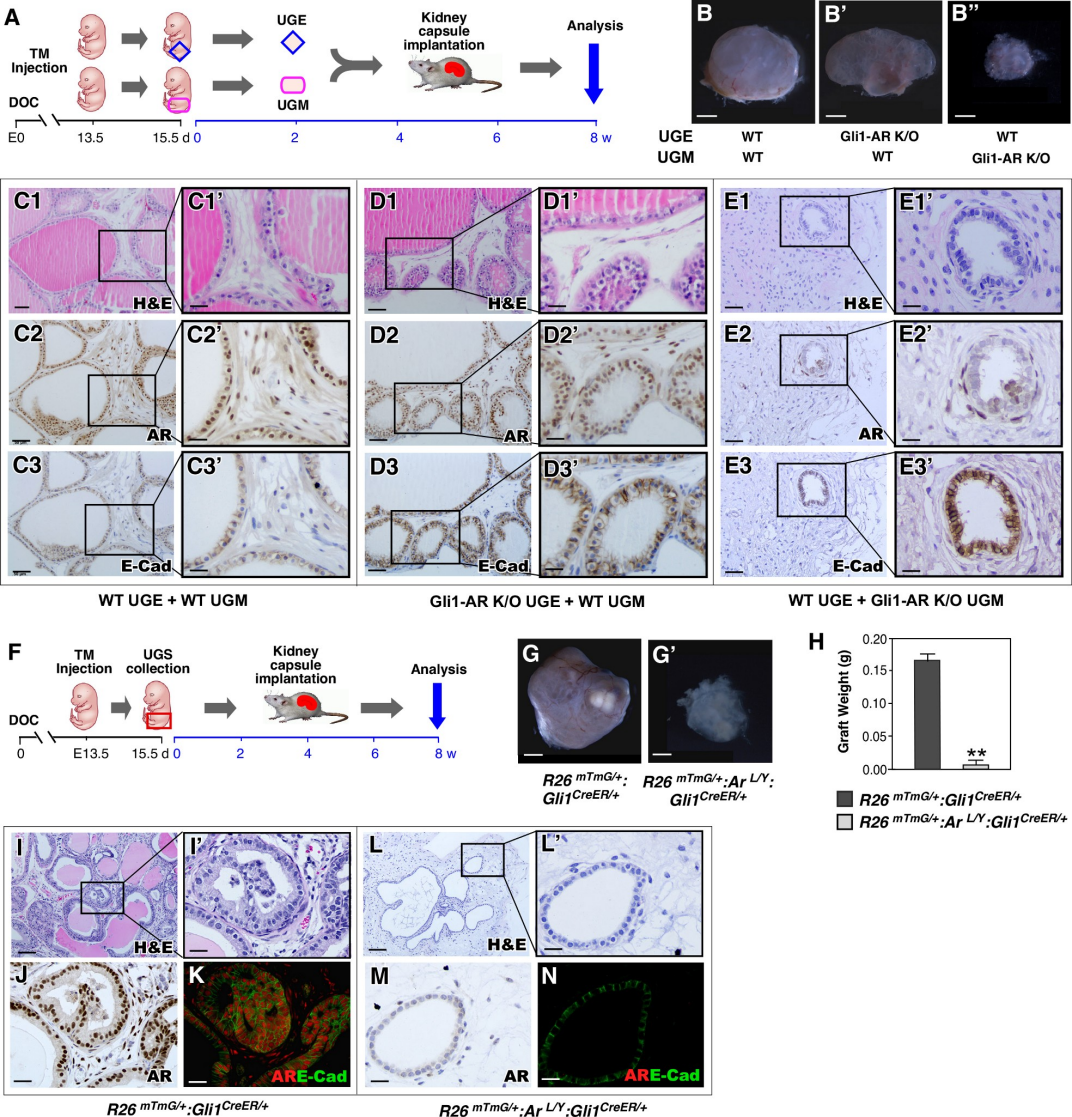
AR expression in mesenchymal Gli1-expressing cells is essential for androgen initiating prostatic budding and prostate gland formation during embryogenesis. ***(A)*** Experimental timeline, including activation of Gli1^CreER^, UGS collection, UGE and UGM dissociation and combination, renal capsule transplantation, and analysis. ***(B)*** Gross images of xenografts from the indicated UGE and UGM combinations. Scale bars, B-B”, 1 mm. ***(C1-E3’)*** Images of H&E, AR, and E-cadherin IHC stained xenograft tissue sections from the indicated UGE and UGM combinations. Scale bars, C1-3, D1-3 and E1-3, 50 μm; C1’-3’, D1’-3’ and E1’-3’, 20 μm. ***(F)*** Experimental timeline, including activation of Gli1^CreER^, UGS collection, renal capsule transplantation, and analysis. ***(G)*** Gross images of implanted grafts from different genotype UGS tissues. Scale bars, G-G’, 1 mm. ***(H)*** Graphical representation of xenografts weight from E15.5 UGS tissue isolated from *R26*^*mTmG/+*^:*Gli1*^*CreER/+*^ or *R26*^*mTmG/+*^:*Ar*^*L/Y*^:*Gli1*^*CreER/+*^ embryos. Error bars indicate s.d. **P < 0.01; analyzed using 2-tailed *t*-test; n = 4 mice per genotype. ***(I-N)*** Images of H&E staining, AR immunohistochemistry, and co-immunofluorescent staining of AR (red) and mGFP (green) of xenograft tissue sections from UGS of mice of the indicated genotypes. Scale bars, I and L, 100 μm; I’, J, K, L’, M and N, 25 μm.

Previous tissue recombination studies showed recombining the UGM of Tfm/Y mice with wild-type UGE failed to yield normal prostate epithelium [3–5], suggesting an essential role of mesenchymal AR signaling in prostate early development. However, the identity of the cell lineages that are responsive to mesenchymal androgen signaling is still unknown. Here, we directly assessed the role of AR in Gli1-expressing cells using similar tissue recombination assays as described previously [3–5]. Grafts recombining UGE and UGM tissues isolated from either *R26*^*mTmG/+*^:*Ar*^*L/Y*^:*Gli1*^*CreER/+*^ or wild-type controls were implanted under the kidney capsule of SCID mice supplemented with androgen pellets and analyzed 8 weeks later (Fig 3A). Grafts combining either wild-type or AR-deficient UGE with wild-type UGM appeared transparent and were larger and heavier than grafts combining wild-type UGE with AR-deficient UGM (Fig 3B, 3B’ versus 3B”). Histologically, control grafts showed a glandular epithelium resembling secretory prostatic glands (Fig 3C1–3C1’ and 3D1–3D1’) and containing typical prostatic luminal cells with positive AR and E-cadherin staining (Fig 3C2–3C3’ and 3D2-D3’). However, grafts composed of wild-type UGE and AR-deficient UGM failed to develop normal prostatic epithelium and had only a few undeveloped glands (Fig 3E1–3E1’), which showed E-cadherin staining but no or very weak AR staining (Fig 3E2–3E2’ and 3E3–3E3’). These results demonstrate that mesenchymal AR expression in Gli1-expressing cells is essential for prostatic epithelial budding and gland formation.

A previous study reported that SHH-deficient mouse embryos develop prostatic defects due to insufficient androgens [19]. To determine if androgen insufficiency contributes to the above prostate abnormalities, we implanted UGS tissues from *R26*^*mTmG/+*^:*Ar*^*L/Y*^:*Gli1*^*CreER/+*^ and *R26*^*mTmG/+*^:*Gli1*^*CreER/+*^ E15.5-old embryos under the kidney capsules of SCID mice supplemented with androgen pellets (Fig 3F). After eight weeks, gross analysis showed that AR-deficient UGS grafts were significantly smaller than those from controls despite having been implanted in the same host with supplemental androgen pellets (Fig 3G’ versus 3G, and 3H). Histologically, AR-deficient UGS showed an abnormal glandular structure in comparison to control counterparts (Fig 3L–3L’ versus 3I–3I’). IHC and co-IF analyses showed the nuclear AR staining in *R26*^*mTmG/+*^:*Gli1*^*CreER/+*^ UGS tissues (Fig 3J and 3K). In contrast, no clear AR staining was revealed in *R26*^*mTmG/+*^:*Ar*^*L/Y*^:*Gli1*^*CreER/+*^ UGS tissues (Fig 3M and 3N). These results demonstrate that selective deletion of AR expression in mesenchymal Gli1-expressing cells directly contributes to abnormal prostate development during embryogenesis, rather than insufficient androgens.

### Deletion of AR expression in prepubescent Gli1-expressing cells rather than insufficient androgens directly leads to prostate growth defects in adulthood

**Fig 4:**
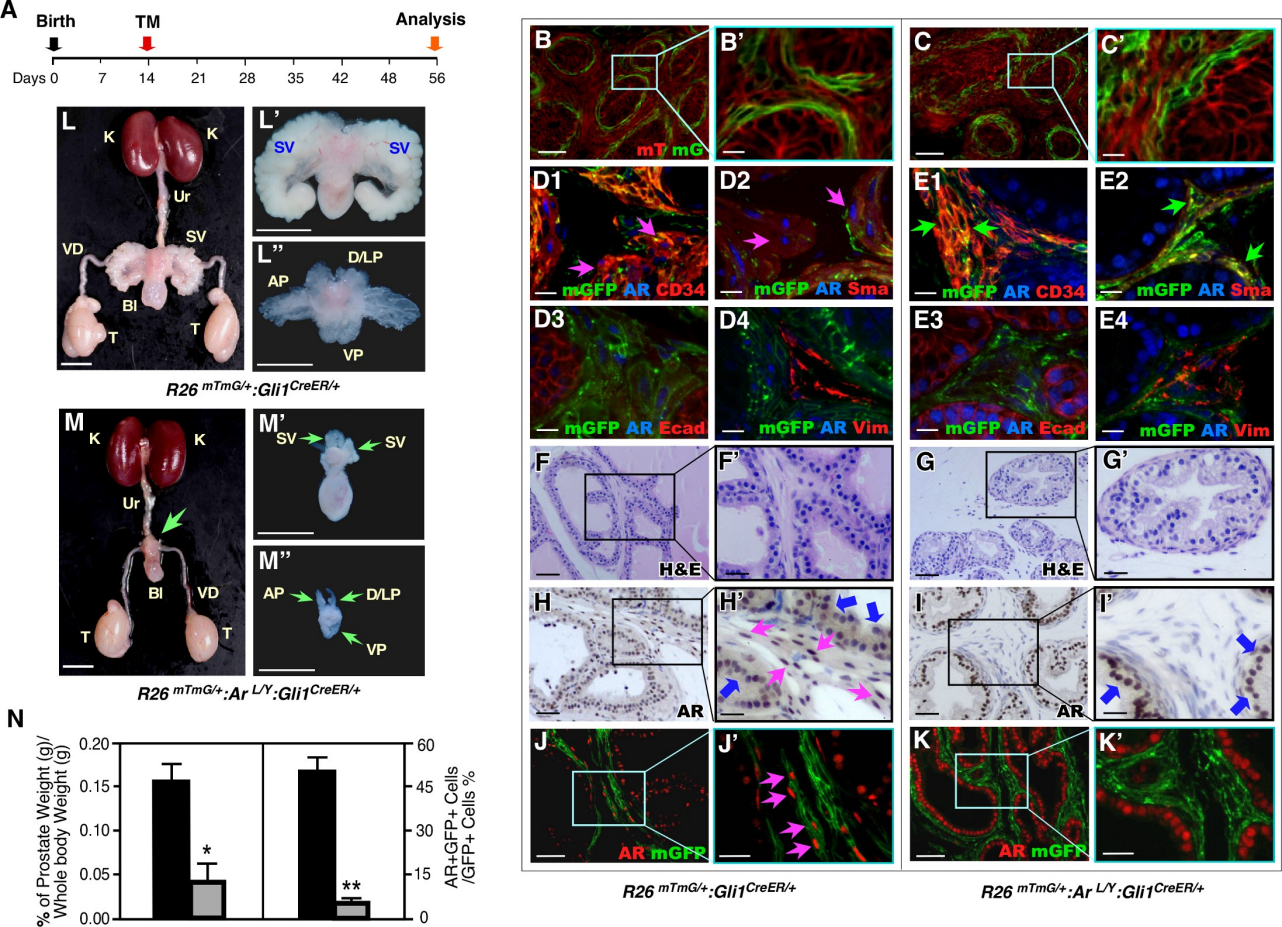
Conditional deletion of AR in pre-pubescent Gli1-expressing cells significantly impairs prostate development and regeneration. *(****A****)* Schematic of experimental timeline for TM induction and analysis. *(****B-C’****)* Fluorescence micrographs of mT and mG expression in P17 prostate tissues from male mice of the indicated genotypes. Scale bars, B, C, 50 μm; B’, C’, 10 μm. *(****D-E****)* Triple-immunofluorescent analyses, using the indicated antibodies, of P56 prostate tissues from male mice of the indicated genotypes. Pink arrows indicate triple positive cells. Green arrows indicate loss of AR expression in GFP and CD34 or SMA double positive cells. Scale bars, 10 μm. *(****F*** *to* ***K’****)* H&E, IHC, and IF analyses of P56 prostate tissues from male mice of the indicated genotypes. Blue arrows indicate epithelial AR-positive cells. Pink arrows indicate stromal AR-positive cells. Scale bars, F-K, 50 μm; F’-K’, 25 μm. *(****L-M****)* Representative urogenital tracts isolated from indicated mice. Green arrows indicate abnormal and undeveloped prostatic lobes. Scale bars; L and M, 1 cm; L’, L”, M’, and M”, 5 mm. *(****N****)* Ratio of prostate wet weight versus whole body weight as percentages (left panel) and quantification of AR and mGFP double positive cells per mGFP-positive cells in mice of the indicated genotypes, n = 6 mice per genotype. Error bars indicate s.d. Detailed information for the quantification is included (S2 Table); * *P* < 0.05, **P < 0.01; analyzed using 2-tailed *t*-test.

An essential role of androgen and SHH signaling has been shown in pubertal and adult prostate [10,11,13]. Therefore, we explored the function of the AR in Gli1-expressing cells during prostate pubertal and adult epithelium growth by administering TM to mice at postnatal day 14 (P14) (Fig 4A). In *R26*^*mTmG/+*^:*Ar*^*L/Y*^:*Gli1*^*CreER/+*^ and *R26*^*mTmG/+*^:*Gli1*^*CreER/+*^ mice, TM-induced mGFP expression was present exclusively in stromal cells surrounding epithelial glands in prostatic tissues at P17 (Fig 4B–4B’ and 4C–4C’) and P56 (S2A–S2B’ Fig). Co-expression of SMA or CD34 with AR and Gli1-driven GFP expression was also observed, indicating the stromal cell properties of Gli1-expressing cells, as reported earlier [13] (Fig 4D1-4 and 4E1-4 and S2C–S2J Fig). Gross examination of 8-week-old *R26*^*mTmG/+*^:*Ar*^*L/Y*^:*Gli1*^*CreER/+*^ male mice showed severe prostate growth defects. All prostatic lobes in these mice were significantly smaller, both in size and weight, than those of control littermates (Fig 4M–4M” versus 4L–4L”, and left panel, Fig 4N). Histological analyses revealed pathologic changes consistent with growth retardation in the mutant mice, which had fewer and smaller prostatic glands than controls (Fig 4G–4G’ versus 4F–4F’). IHC or co-IF analyses showed a decrease in AR expression in the prostatic stromal compartment (Fig 4I–4I’ and 4K–4K’) or in GFP-positive cells in mutant mouse prostate tissues, respectively, as compared to controls (Fig 4H–4H’ and 4J–4J’ and 4N, right panel). We saw no change in epithelial AR expression in samples from both AR-deficient and control mice. Together, these results shed fresh light on the role of mesenchymal AR rather than epithelial AR signaling in promoting prepubescent prostate growth and morphogenesis. They are consistent with results observed in the embryonic UGS tissues and further emphasize an indispensable role for mesenchymal AR in Gli1-expressing cells in regulating prostatic epithelial morphogenesis and growth.

**Fig 5:**
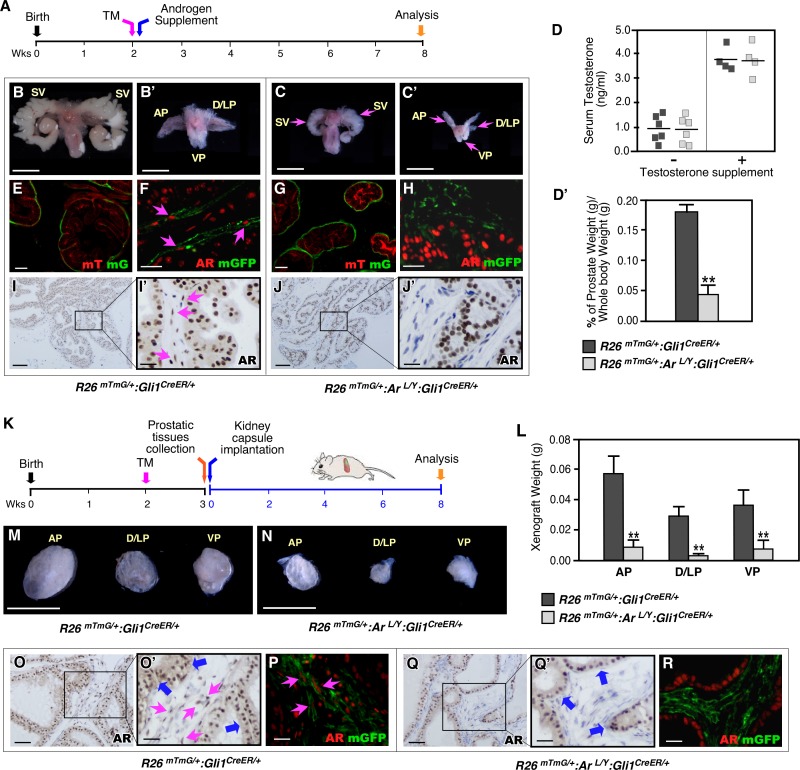
Deletion of AR expression in Gli1-expressing cells rather than insufficient androgen results in severe prostate defects. ***(A)*** Experimental timeline including activation of *Gli1*^*CreER*^, administration of androgen supplement, and analysis. ***(B-C)*** Representative images of urogenital tissue isolated from *R26*^*mTmG/+*^:*Ar*^*L/Y*^:*Gli1*^*CreER/+*^, and *R26*^*mTmG/+*^:*Gli1*^*CreER/+*^ mice. Scale bars, B-C’, 1 mm. ***(D-D’)*** Serum testosterone concentrations and percentage of prostate wet weight versus whole body weight of mice of the indicated genotypes. Error bars indicate s.d. **P < 0.01; analyzed using 2-tailed *t*-test. ***(E*,*G)*** mTomato or mGFP expression in androgen supplemented P56 prostates from *R26*^*mTmG/+*^:*Gli1*^*CreER/+*^ and *R26*^*mTmG/+*^:*Ar*^*L/Y*^:*Gli1*^*CreER/+*^ mice. Scale bars, 50 μm. ***(F*,*H)*** Co-localization of AR and mGFP proteins in prostate tissues isolated from mice of the indicated genotypes. Pink arrows indicate AR-expressing mGFP-positive cells. Scale bars, 20 μm. ***(I*,*J)*** AR expression (brown) in prostate tissues of *R26*^*mTmG/+*^:*Gli1*^*CreER/+*^ and *Ar*^*L/Y*^:*Gli1*^*CreER/+*^ or *R26*^*mTmG/+*^:*Ar*^*L/Y*^:*Gli1*^*CreER/+*^ mice. Pink arrows indicate stromal AR-positive cells. Scale bars, I-J, 100 μm; I’-J’, 20 μm. ***(K)*** Experimental timeline including activation of *Gli1*^*CreER*^, prostate tissue collection, renal capsule transplantation, and analysis. ***(L-N)*** Graphical representation of the weight and gross images of xenografts grown from prostatic lobes of *R26*^*mTmG/+*^:*Gli1*^*CreER/+*^ or *R26*^*mTmG/+*^:*Ar*^*L/Y*^:*Gli1*^*CreER/+*^ mice. Scale bars, M-N, 1mm. ***(O*,*Q)*** AR expression (brown) in xenografted tissues from the indicated genotypes. Blue arrows indicate epithelial AR-positive cells, pink arrows indicate stromal AR-positive cells. ***(P*,*R)*** Localization of AR (red) and mGFP (green) in xenograft tissues isolated from the indicated genotypes. Pink arrows indicate AR-expressing mGFP-positive cells. Scale bars, O, Q, 50 μm; O’, P, Q’, and R, 25 μm.

Given the earlier study showing that prostatic defects in SHH-deficient mouse embryos were due to insufficient levels of androgens [19], we performed a series of experiments to further determine whether AR activation in Gli1-expressing cells or insufficient androgen levels plays a role in resulting in the prostate defects as we observed above. Both AR-deficient mice and normal littermates were supplemented with androgen pellets (Fig 5A). Despite the fact that these mice showed higher levels of serum androgens than their counterparts without supplementation (Fig 5D), similar prostate growth defects including smaller and lighter prostate lobes were revealed in AR-deficient mice but not in supplemented control littermates (Fig 5B–5B’ versus 5C–5C’, and 5D’). Histological analyses also showed fewer and smaller prostate glands in androgen supplemented AR-deficient mice than controls (S3G–S3L’ Fig). Gli1-driven mGFP expression was revealed within prostatic mesenchymal cell compartments in both AR-deficient and control mice that received androgen supplementation (Fig 5E and 5G). Both co-IF and IHC assays showed a significant reduction of AR expression in prostatic Gli1-driven GFP-expressing cells and stromal cells in AR-deficient prostatic tissues in comparison to control samples (Fig 5H versus 5F, and Fig 5J–5J’ versus 5I–5I’). We then further determined the potential effect of insufficient androgens on the above prostate defects by implanting prostate tissues isolated from AR-deficient and control littermates under the kidney capsules of SCID mice supplemented with androgen pellets (Fig 5K). Eight weeks after implantation, the AR-deficient grafts appeared smaller and weighed less than control littermate grafts implanted in the same hosts (Fig 5N versus 5M and 5L). Histologically, AR-deficient implant tissues also revealed retarded growth, featuring fewer, smaller prostate glands (S3M–S3R’ Fig). GFP-positive cells appeared in both AR-deficient and control implants (Fig 5P and 5R). Substantially fewer AR-positive cells were present in stromal compartments of AR-deficient tissues than of controls (pink arrows, Fig 5O–5O’, and 5P versus 5Q-Q’ and 5R) while AR-expression in epithelial compartments was comparable across tissue samples (blue arrows, Fig 5O’ and 5Q’). Through the above experiments, we determined the critical role of AR in Gli1-positive stromal cells in regulating prostate growth and morphogenesis during puberty, and ruling out the potential role of insufficient androgens in the above prostatic abnormalities.

### Deletion of AR expression in mesenchymal Gli1-expressing cells impairs prostatic epithelium regeneration

**Fig 6:**
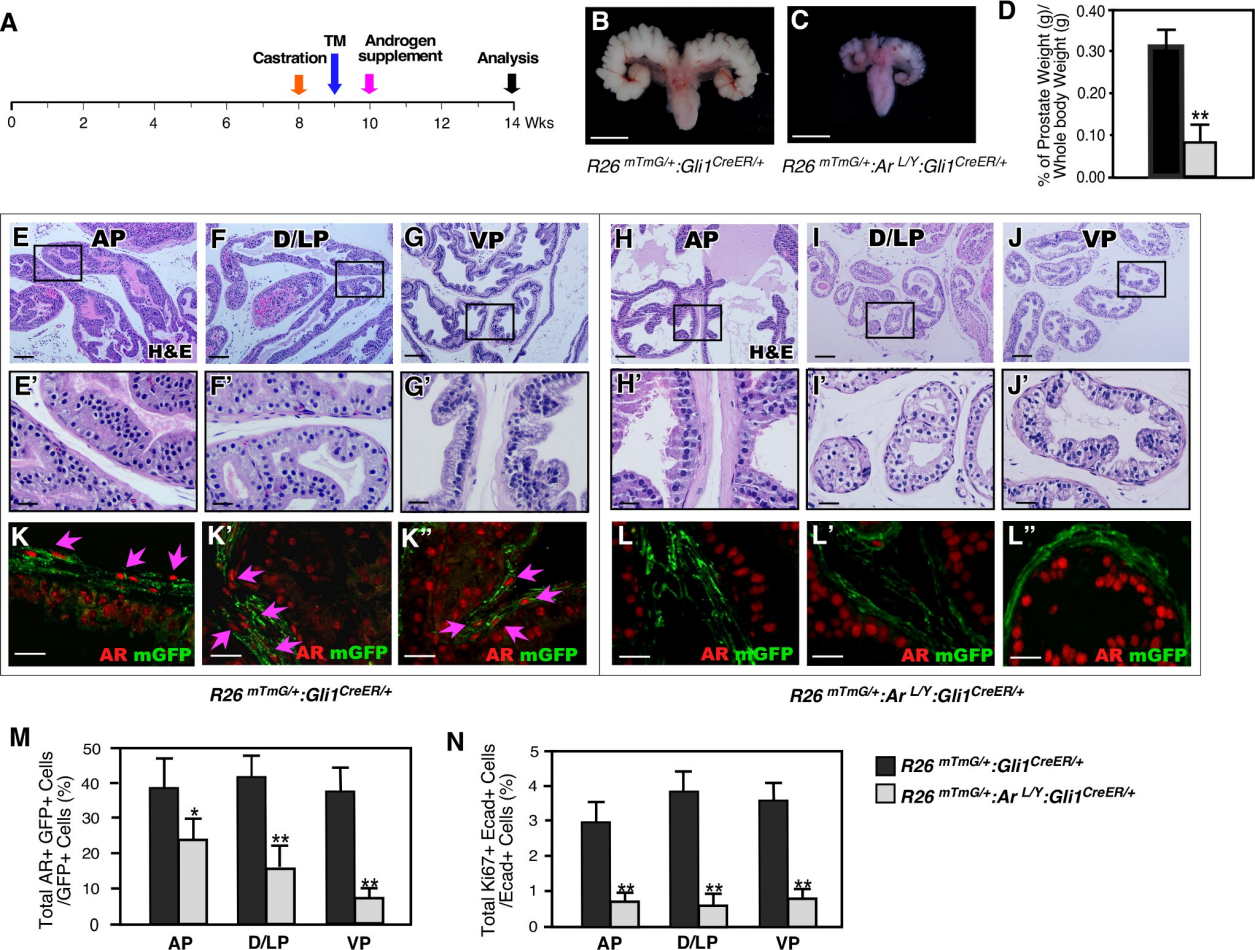
Deletion of AR in Gli1-expressing cells after castration reduces their regenerative ability in adult prostates. ***(A)*** Experimental timeline for castration, activation of Gli1^CreER^ by TM injection, androgen supplementation (regeneration), and analysis. ***(B*, *C)*** Gross images of seminal vesicles and prostatic tissues from different genotype mice as indicated in images. Scale bars, 1 mm. ***(D)*** Graphical representation of the ratio of prostate wet weight versus whole body weight of regenerated prostates isolated from *R26*^*mTmG/+*^:*Gli1*^*CreER/+*^ or *R26*^*mTmG/+*^:*Ar*^*L/Y*^:*Gli1*^*CreER/+*^ mice. Error bars indicate s.d. **P < 0.01; analyzed using 2-tailed *t*-test, n = 4 per genotype. ***(E-J’)*** Representative H&E stained tissue sections from different prostatic lobes of *R26*^*mTmG/+*^:*Gli1*^*CreER/+*^ and *R26*^*mTmG/+*^:*Ar*^*L/Y*^:*Gli1*^*CreER/+*^ mice. Scale bars, E-J, 100 μm; E’-J’, 25 μm. ***(K-L”)*** Co-localization of mGFP (green) and AR (red) from different prostatic lobes of regenerated prostates from mice of the indicated genotypes. Scale bars, 20 μm. ***(M-N)*** Quantification of AR-mGFP double positive cells per mGFP-positive cells and Ki67-E-cadherin double positive cells per E-cadherin positive cells from different prostatic lobes of regenerated prostate tissue from *R26*^*mTmG/+*^:*Gli1*^*CreER/+*^ (black bars) and or *R26*^*mTmG/+*^:*Ar*^*L/Y*^:*Gli1*^*CreER/+*^ (grey bars) mice. Error bars indicate s.d, *: *P* < 0.05; **: *P* <0.01; analyzed using 2-tailed *t*-test, n = 4 per genotype (Detailed information for the quantification is included in S3 & S4 Tables).

Previous work has shown that Gli1-expressing cells have the regenerative capability to repopulate prostatic stroma [13]. We thus assessed the role of stromal Gli1-expressing cells in regeneration of prostatic epithelium. Both AR-deficient and wild type mice were castrated and subsequently underwent regeneration by implantation of androgen pellets (Fig 6A). Gross examination of prostate tissues that underwent 4 weeks of regeneration revealed that those from AR-deficient mice were significantly smaller and weighed less than those from control littermates (Fig 6B–6D), and histologically, they also showed fewer and smaller epithelial ducts in all four prostatic lobes than those of control littermates (Fig 6H–6J’ versus 6E–6G’). Decreased AR positive cells appeared in stromal compartments of regenerated prostates in AR-deficient mice as compared to those of controls (S4B–S4B” Fig versus S4A–S4A” Fig). Co-IF showed very few AR and GFP double positive cells (Fig 6L–6L” versus 6K–6K”, and 6M), indicating selective deletion of AR in Gli1-expressing cells. In addition, fewer Ki67+ prostatic epithelial cells were present in AR-deficient mice (Fig 6N, S4D–S4D” versus S4C–S4C” Fig). These data are consistent with our previous observations and suggest a critical role of stromal Gli1-expressing cells in androgen-induced epithelial cell regeneration in addition to their role in repopulating prostate stroma.

### Mesenchymal Gli1-expressing cells regulate prostate epithelium growth through androgen-induced reciprocal paracrine pathways

**Fig 7:**
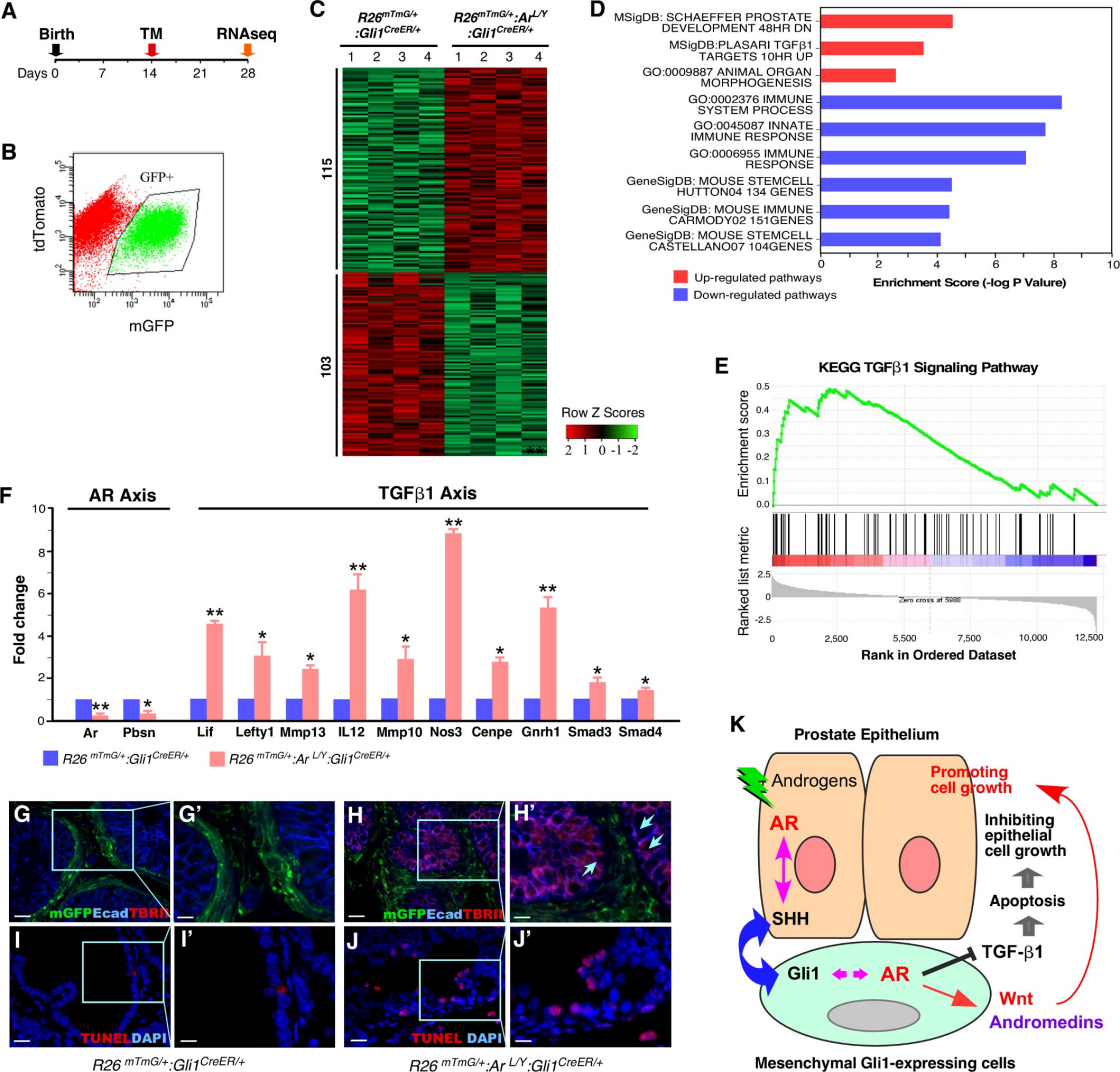
Identifying molecular mechanisms by which loss of AR in Gli1-expressing cells dysregulates prostate growth. *(****A****)* Schematic for the activation and collection of Gli1-expressing cells from AR mutant mice and controls for RNA-sequencing. *(****B****)* Representative analysis of FACS-sorted GFP-labeled prostatic stromal cells. The purified GFP positive cells (>99%) were used in the experiments. *(****C****)* Heatmaps showing differentially expressed genes (DEGs) between mGFP-positive cells isolated from *R26*^*mTmG/+*^:*Gli1*^*CreER/+*^ and *R26*^*mTmG/+*^:*Ar*^*L/Y*^:*Gli1*^*CreER/+*^ mice (1.5-fold difference; false discovery rate [FDR] < 0.05). Row Z scores as determined using Bioconductor edgeR software (S5 Table). *(****D****)* Pathway analysis mapping genes to MSigDB and GO (DAVID) terms, enrichment scores were calculated [-log10 (P-value)]. Representative pathways that were significantly up-regulated (red) or down-regulated (blue) (*P* < 0.05) are shown. *(****E****)* GSEA showed significant positive association with TGFβ1 from DEGs that were differentially expressed between the mutant and control groups (FDR < 0.05, pre-ranked GSEA). KEGG pathway TGFβ1 (NES = 1.76, FDR < 0.031). *(****F****)* Fold change in expression of genes from the AR and TGFβ1 axes determined by qRT-PCR analysis of FACS-sorted cells from *R26*^*mTmG/+*^:*Gli1*^*CreER/+*^ or *R26*^*mTmG/+*^:*Ar*^*L/Y*^:*Gli1*^*CreER/+*^ mice. Error bars indicate s.d.; *P < 0.05, ** *P* < 0.01; analyzed using 2-tailed students’ *t* test. (n = 3 replicates per data point). *(****G-H’****)* Co-IF staining for mGFP, E-cadherin, and TGFβRII in P56 prostate tissues isolated from mice of the indicated genotypes. Blue arrows denote TGFβRII-expressing epithelial cells adjacent to Gli1-derived GFP-positive cells, ***H’***. Scale bars, G and H, 20 μm; G’ and H’, 10 μm. (**I-J**) TUNEL assay of prostate tissues isolated from mice of the indicated genotypes. Blue arrows indicate apoptotic cells. Scale bars, I and J, 20 μm; I’ and J’, 10 μm. *(****K****)* Schematic of suggested molecular mechanism by which stromal AR signaling in SHH effector, Gli1-expressing cells regulates development of the prostatic epithelium through TGFβ1 and other signaling pathways.

To gain mechanistic insights into how AR signaling in stromal Gli1-expressing cells regulates prostate morphogenesis and growth, we performed RNA-sequencing (RNA-seq) analyses using RNA samples isolated from prostatic GFP-positive cells isolated from both *R26*^*mTmG/+*^:*Gli1*^*CreER/+*^ and *R26*^*mTmG/+*^:*Ar*^*L/Y*^:*Gli1*^*CreER/+*^ mice (Fig 7A and 7B). We identified 218 statistically significant differentially expressed genes (DEGs), among which 115 genes were up-regulated and 103 genes were down-regulated in AR-deficient Gli1-expressing cells from *R26*^*mTmG/+*^:*Ar*^*L/Y*^:*Gli1*^*CreER/+*^ mice as compared to normal Gli1-expressing cells from age- and sex-matched *R26*^*mTmG/+*^:*Gli1*^*CreER/+*^ controls (Fig 7C and S5 Table). Using Gene Set Enrichment Analysis (GSEA) [20], we identified aberrant signaling pathways in AR-deficient Gli1-expressing cells. Alterations in signaling pathways related to prostate development, stem cells, and organ morphogenesis appeared to show significant changes (Fig 7D). Specifically, the transforming growth factor β1 (TGFβ1) pathway was up-regulated in AR-deficient Gli1-expressing cells (Fig 7D). Consistent with this, significant enrichment of the TGFβ1 pathway was observed in DEGs between AR-deficient Gli1-expressing cells versus controls (Fig 7E). An inhibitory effect of stromal TGFβ1 signaling on prostate epithelium growth has been suggested [21,22], but the molecular mechanism for this mesenchymal-epithelial regulation remains unclear. Our observation of increased TGFβ1 signaling in AR-deficient Gli1-expressing cells suggests that stromal androgen signaling may attenuate the inhibitory effect of TGFβ1 signaling in prostate epithelial growth and regeneration.

To address this hypothesis, we performed a series of proof-of-principle experiments. Using quantitative reverse transcription-PCR (qRT-PCR), we observed reduced expression of AR transcripts and its downstream target, probasin, in AR-deficient Gli1-expressing cells (Fig 7F). While the expression of probasin in the stromal Gli1-expressing cells is significantly lower than in prostatic epithelial cells, consistent with previous reported data [23], a reduction in probasin expression was revealed in Gli1-expressing cells (S5A Fig). Importantly, elevated expression of TGFβ1 downstream targets and effectors were revealed in the same AR-deficient cells (Fig 7F), suggesting a repressive role of androgen signaling on the TGFβ1 pathway. Increased expression of the TGF-βII receptor, an effector of the TGFβ1 signaling pathway, appeared in epithelial cells adjacent to Gli1-driven mGFP-positive cells in AR-deficient prostate tissues (blue arrows, Fig 7G and 7H). Increased apoptosis in prostatic epithelial cells was also present within the same AR-deleted samples (Fig 7I and 7J). Data from the above proof-of-principle experiments suggest a regulatory mechanism for stromal androgen signaling in eliciting prostate epithelial cell growth through mesenchymal-epithelial regulation (Fig 7K).

## Discussion

Prostate embryonic development, subsequent pubertal and adult growth, homeostatic maintenance, and regenerative capability require androgen-mediated paracrine interactions between stromal and epithelial cell compartments [1,2] [6]. The expression of AR occurs in urogenital mesenchyme before and during prostate morphogenesis, and then extends to urogenital epithelia after prostatic budding and the beginning of branching morphogenesis[24,25]. Earlier tissue recombinant studies demonstrated that mesenchymal AR, but not epithelial AR, is essential for prostatic epithelial development and morphogenesis [1,3]. However, despite significant effort over the past three decades, the cellular identity of the prostatic mesenchymal cells that are responsive to androgen induction and stimulate UGE morphogenesis and development is still unknown. In this study, we identify for the first time an indispensable role of AR in mesenchymal Gli-1 expressing cells in regulating early prostate development, morphogenesis, and regeneration. Specifically, using classical tissue recombinant assays, we demonstrated that UGM tissues with AR-deficient Gli1-expressing cells fail to facilitate prostatic gland formation from wild type UGE, similar to previous studies with Tfm UGM and wild type UGE tissues. These data demonstrate a novel role of mesenchymal Gli1-expressing cells in androgen signaling-mediated prostatic budding and gland formation during embryogenesis. In addition, using mouse genetic tools, we further showed the critical role of the AR in prostatic Gli1-expressing cells during the course of pubertal and adult growth, morphogenesis, and regeneration. These data provide scientific evidence demonstrating that androgen signaling in mesenchymal Gli1-expressing cells is essential for inducing prostatic epithelium development, growth and renewal in response to androgen signaling. Our findings not only determine the critical roles of the androgen and SHH signaling pathways in regulating prostatic cell fate, growth, and renewal through paracrine regulation, but also shed fresh light on a long-standing question regarding the properties of mesenchymal androgen-responsive cells that are primarily responsible for eliciting embryonic prostate development. Additionally, the fundamental principles underlying sex hormone and SHH signaling interactions in mesenchymal cells described here may be applicable to the development, morphogenesis and regeneration of other reproductive organs and tissues. Thus, the current mouse models will be useful for more in-depth mechanistic studies to address those important questions in the future.

SHH signaling plays a critical role in prostatic development, homeostasis, and tumorigenesis through reciprocal epithelial-mesenchymal interactions [11,26]. Prostatic Gli1-expressing cells have been implicated to possess stromal stem/progenitor cell properties with the ability to repopulate prostatic stromal cells during androgen depletion and supplementation cycles [13]. However, an earlier study has shown that Gli1 is dispensable for mouse prostate development and morphogenesis [12]. In this study, we demonstrate an indispensable role of AR expression in prostatic Gli1-expressing cells during prostate development, growth, and regeneration. Our findings suggest a novel mechanism through which mesenchymal androgen and SHH signaling pathways control prostatic epithelial cell fate, morphogenesis, and renewal. These data also explore the potential role of these Gli1-expressing cells acting as a cellular niche for prostatic epithelial stem- and progenitor-induced cell differentiation, growth, and regeneration in the prostate. Further studies on the regulation of androgen signaling in Gli1-expressing cells may provide new insight into our knowledge regarding stromal androgen signaling and the interaction with SHH signaling pathways in the prostate.

To gain more insight into molecular mechanisms underlying AR action in Gli1-expressing cells, we performed RNA-sequencing analyses with samples isolated from either wild type or AR-deficient Gli1-expressing cells in mouse prostate tissues. We identified aberrant alterations in signaling pathways related to prostate development, stem cells, organ morphogenesis in AR-deficient Gli1-expressing cells. Specifically, the transforming growth factor β1 (TGFβ1) pathway was up-regulated in AR-deficient Gli1-expressing cells. Consistent with this, significant enrichment of the TGFβ1 pathway was observed in DEGs between AR-deficient Gli1-expressing cells. We further performed a series of proof-of-principle experiments to demonstrate that elevated TGFβ1-mediated pathways in AR-deficient mesenchymal Gli1-expressing cells inhibit the growth of prostate epithelium through mesenchymal-epithelial paracrine regulation. We observed elevated expression of TGFβ1 downstream targets and effectors in AR deficient Gli1-expressing cells at embryonic stages and postnatal days 28 and 56, respectively (Fig 7F and S5B and S5C Fig). Since previous studies have shown an inhibitory effect of TGFβ1 signaling on prostate epithelium growth through paracrine regulation [21,22], our data provide mechanistic insight into how mesenchymal androgen signaling in Gli1-expressing cells can regulate prostatic epithelial growth via inducing TGFβ1 mediated cell growth arrest and apoptosis of prostate epithelium [27,28]. In this study, we also observed aberrant alterations of other signaling pathways related to prostate development, stem cells, and organ morphogenesis in AR deficient Gli1-expressing cells. It has been shown that a variety of andromedins are produced in mesenchymal AR expressing cells and play significant and different roles in inducing both epithelial and stromal early development and morphogenesis [6,29]. Therefore, further in-depth mechanistic analyses using the current mouse models are extremely important for determining the signaling pathways and master regulators in facilitating the biological roles of AR in stromal Gli1-expressing cells in prostate development, morphogenesis, and regeneration.

## Materials and methods

### Ethics statement

All experimental procedures and care of animals in this study were carried out according to the Institutional Animal Care and Use Committee (IACUC) at Beckman Research Institute at City of Hope, and approved by the IACUC. Euthanasia was performed by CO2 inhalation followed by cervical dislocation.

### Mouse experiments

*Gli1*^*CreER*^ and *ROSA*^*mTmG*^ mice were obtained from Jackson Laboratories (stocks 18867 and 7676). *Ar*^*Lox/Y*^ mice were obtained from Dr. Guido Verhoeven [30]. To elicit genetic recombination, mice were intraperitoneally injected with 125 μg/g body weight of tamoxifen (TM; Sigma) suspended in corn oil (Sigma) [31,32]. To label Gli1-expressing cells in embryos, pregnant females were given a single intraperitoneal injection of TM (125 μg/g body weight). For the prostate regeneration study, three consecutive daily intraperitoneal injections of 200 μg/g body weight of TM (injected on P63-P65) were given.

Adult male mice were castrated as described previously [33]. For androgen supplementation, testosterone pellets (12.5 mg, Innovative Research of America) were placed subcutaneously in the backs of the mice. To prepare postnatal prostatic tissues for kidney capsule transplantation, mice were injected with TM on P14 [31,32]. On P21, the mice were euthanized and their prostates collected. Individual prostatic lobes were separated and kept in DMEM (Gibco), 5% FBS (HyClone), and 1% Penicillin/Streptomycin (Gibco) while SCID mice were prepared for the kidney capsule transplantation procedure as described previously [31,32]. For tissue recombination assays, pregnant females were injected with TM (125 μg/g body weight) at E13.5 and euthanized on E15.5. The UGE and UGM were separated by treatment with 1% trypsin (Gibco) at 4°C for 90 min, followed by mechanical dissociation. Various combinations of UGE and UGM were made as indicated in Fig 3. Dissociated UGM and UGE were combined on 0.4% agar plates containing DMEM with 10% FBS supplementation, followed by incubation at 37°C for overnight. The combined tissues were then implanted under the kidney capsule of 8-week-old male SCID mice the following day, and the grafts were analyzed 8 weeks later.

### Histology and immunostaining

Prostate tissues were fixed in 10% neutral-buffered formalin (American Master Tech Scientific) and processed into paraffin. Five-micron serial sections were cut and processed from Clearify (American MasterTech Scientific) to PBS through a decreasing ethanol gradient. For histological assessment, hematoxylin and eosin staining was performed as described [31,32]. For IHC, slides were treated by boiling in 0.01 M citrate buffer (pH 6.0) for antigen retrieval, incubated in 0.3% H_2_O_2_ for 15 min, blocked in 5% normal goat serum (Gibco) for 1 h, and incubated with appropriate antibodies (see S6 Table) diluted in 1% normal goat serum at 4°C overnight. Slides were then incubated with biotinylated secondary antibodies for 1 h followed by horseradish peroxidase streptavidin (Vector Laboratories) for 30 min and visualized using a DAB kit (Vector Laboratories). Slides were counterstained with 5% (w/v) Harris Hematoxylin (Thermo Scientific), and coverslips were mounted. For detecting membrane-bound Tomato (mT) and membrane-bound green fluorescent protein (mGFP) signals, tissues were fixed in 10% neutral-buffered formalin at 4°C overnight, cryoprotected in 30% sucrose at 4°C overnight, and embedded in OCT (Tissue-Tek). Five-micron sections on slides were washed three times with PBS. To detect mT and mGFP signals, slides were directly mounted using VECTASHIELD Mounting Medium with DAPI (Vector Laboratories). For IF staining, slides were treated for antigen retrieval as described above, blocked in 5% normal goat serum for 1 h, and incubated with primary antibodies diluted in 1% normal goat serum at 4°C overnight. Slides were washed in PBS then incubated with fluorescent-conjugated secondary antibodies for 1 h, and then mounted as described above. Detailed information regarding antibodies that were used in this study was provided (S6 Table).

### Microscope image acquisition

Hematoxylin and eosin and immunohistochemistry slides were imaged using an Axio Lab A1 microscope with 10x and 40x Zeiss A-Plan objectives. Images were taken with a Canon EOS 1000D camera and analyzed using Axiovision software (Carl Zeiss). Images of immunofluorescent staining and mTmG signals were acquired on an Olympus Motorized Inverted Research Microscope Model IX81 using 20x and 40x Olympus Plan Fluor objectives, a QImaging RETIGA 2000R camera, and Image-Pro 6.3 software (Media Cybernetics).

### Serum testosterone measurement

Mouse serum testosterone levels were measured using a Mouse/Rat Testosterone ELISA kit (Alpco Diagnostic). Mouse blood samples were collected and tested following the manufacturer’s protocol. The concentration of each sample, corresponding to mean of absorbance value, was then calculated from the calibration curve.

### Preparation of dissociated GFP-expressing cells

To prepare Gli1-expressing cells for RNA sequencing, male mice from different genotypes were injected with TM (1 mg) on P14 or TM (125 μg/g body weight) to activate deletion of AR in Gli1-positive cells. On day E16.5 or P28 or P56, the mice were euthanized and their UGS or prostate tissues were collected, minced into small pieces, and digested with 1 mL of collagenase (10 mg/mL, StemCell Technologies) in DMEM with 10% FBS, DHT (10 nM) and Y-27632 (10 μM) (StemCell Technologies) at 37°C for 90 min. Cells were then digested with TrypLE (1 mL, Gibco) at 37°C for 15 min and were centrifuged at 300xg for 5 min. Cells were passed through 40-μm nylon mesh (Fisherbrand), washed twice with DMEM 10% FBS and dissolved in 500 μL of PBS-2% BSA-1 mg/mL DAPI for cell sorting. Cells were sorted for mGFP-positive and tdTomato-negative, or CD24 positive for prostatic luminal epithelial cells [34]. After sorting, cells were dissolved in DMEM (100 μL) with 10% FBS and counted using Trypan blue (Gibco). Purity of mGFP-positive cells was confirmed by counting the number of mGFP-positive cells compared to total number of cells which stained negative for Trypan blue. All of the samples used in the study possessed >99% purity.

### RNA extraction and RNA-seq libraries preparation and sequencing

RNA samples were extracted with TRIZOL (500 μL, Zymo Research). RNA sequencing libraries were prepared by using SMARTer Ultra Low Input RNA Kit for Sequencing v4 (TaKaRa Clontech) and KAPA Hyper Prep Kit (KAPA Biosystems) according to the manufacturer's protocol. The resulting double stranded cDNA was sheared using a Covaris LE220 Plus (Covaris) with a 200 bp peak in the 50μl volume setting. The fragmented cDNA underwent end repair, 3′ end adenylation and ligation with barcoded adapters. The libraries were validated using the Agilent Bioanalzyer DNA High Sensitivity kit (Agilent), and quantified using the Qubit dsDNA HS Assay Kit (Thermo Fisher Scientific). The sequencing library templates were prepared for sequencing using the Illumina HiSeq SR Cluster V4 Kit. Sequencing runs were performed on an Illumina HiSeq 2500 using the single read mode of 51 cycles of read 1 and 7 cycles of index read with the SBS V4 Kit. Real-time analysis (RTA) 2.2.38 software was used to process the image analysis and base calling.

### Quantitative real-time PCR

qPCR reactions were performed in triplicate using an Applied Biosystems 7900 Fast sequence detector with SYBR Green PCR master mix (Applied Biosystems, Thermo Fisher Scientific). Primers were designed using PrimerQuest (IDT) (S7 Table). All reactions were normalized to expression of the housekeeping gene PP1A. Detailed information regarding primers that were used in this study was provided (S7 Table).

### RNA-seq data processing

RNA sequences were aligned to mouse genome assembly mm9 using TopHat v2 [35], and Ensemble gene expression levels were calculated using HTseq-count [36]. Bioconductor package edgeR [37,38] was used to normalize the data and calculate P value and log2 fold change among groups. The un-clustered heat map was generated using Heatmapper (http://heatmapper.ca/expression/) [39]. To calculate row Z-Score, rows were centered and scaled by subtracting the reads per kilobase of transcript per million mapped reads (RPKM) mean of the row from every RPKM value and then dividing the resulting values by the standard deviation of the row. To compare the unranked list of gene of each cluster versus GO-term-annotated genes [40,41] and the MSigDB annotated genes sets, a P value threshold of P < 0.05 was used. For GSEA, genes were pre-ranked by the signed P value score, which was -log10(P) with the sign of the log2 fold change. Pre-ranked data were uploaded to GSEA and enrichment of Hallmark and KEGG [42] gene sets was interrogated with 1,000 random permutations to obtain the false discovery rate (q value < 0.05) and normalized enrichment score. RNA-seq data is deposited at GEO, under the accession number: GSE140823.

### Statistical analysis

Statistical analyses were performed using GraphPad Prism 6. All data are presented as mean ± s.d. Two group comparisons were analyzed with a two-tailed Student’s t-test and a value of P < 0.05 was taken as statistically significant.

## Supporting information

S1 Fig Histopathologic analysis of postnatal prostates from mice with AR deletion in Gli1-expressing cells during embryogenesis. Representative images of squamous metaplasia ***(A)*** and squamous cysts ***(B)*** with prominent central keratinization from prostate tissues isolated from 8-week old *R26*^*mTmG/+*^:*Ar*^*L/Y*^:*Gli1*^*CreER/+*^ mice. Scale bars, A, B, 200 μm; A’, B’, 20 μm.
(PDF)

S2 Fig Analyses of cellular properties of prostatic Gli1-expressing cells in mouse tissues. ***(A-B)*** Fluorescence micrographs of mT and mG expression from P56 prostate tissues from male mice of the indicated genotypes. Scale bars, A-B, 20 μm; A’-B’, 10 μm. ***(C1 to J5)*** Images of triple-immunofluorescent staining of prostatic tissues with the indicated antibodies. White arrows indicate AR and GFP double positive cells (C5). Pink arrows indicate triple positive cells (D5 and E5). Blue arrows indicate AR-negative, GFP-positive, and E-cadherin negative cells (G5). Green arrows indicate AR-negative, GFP-positive cells with either CD34 (H5) or SMA (I5) staining. Scale bars, C1-4, D1-4, E1-4, F1-4, G1-4, H1-4, I1-4, and J1-4, 20 μm; C5, D5, E5, F5, G5, H5, I5, and J5, 10 μm. ***(K)*** Quantification of AR and E-cadherin double positive cells per E-cadherin-positive cells (left panel) and AR and mGFP double positive cells per mGFP-positive cells (right panel) in P56 prostate tissues from male mice of the indicated genotypes. Error bars indicate s.d., analyzed using 2-tailed *t*-test; n = 6 mice per genotype.
(PDF)

S3 Fig Histologic analysis of prostatic lobes from *R26^mTmG/+^:Gli1^CreER/+^* and *Ar^L/Y^:Gli1^CreER/+^* or *R26^mTmG/+^:Ar^L/Y^:Gli1^CreER/+^* mice. ***(A-F’)*** Representative H&E staining of prostatic lobes from P56 prostates isolated from *R26*^*mTmG/+*^:*Gli1*^*CreER/+*^ or *R26*^*mTmG/+*^:*Ar*^*L/Y*^:*Gli1*^*CreER/+*^ mice. ***(G-L’)*** Representative H&E staining of prostatic lobes from P56 prostates isolated from androgen supplemented *R26*^*mTmG/+*^:*Gli1*^*CreER/+*^ and *R26*^*mTmG/+*^:*Ar*^*L/Y*^:*Gli1*^*CreER/+*^ mice. ***(M-R’)*** Representative H&E staining of 8-week old implants from *R26*^*mTmG/+*^:*Gli1*^*CreER/+*^ or *R26*^*mTmG/+*^:*Ar*^*L/Y*^:*Gli1*^*CreER/+*^ P14 prostatic lobes. Scale bars, A-R 100 μm; A’-R’ 20 μm.
(PDF)

S4 Fig Deletion of AR in Gli1-expressing cells after castration reduces their regenerative ability in adult prostates. ***(A-B”)*** IHC analyses for AR expression in different prostatic lobes of regenerated prostates from *R26*^*mTmG/+*^:*Gli1*^*CreER/+*^ or *R26*^*mTmG/+*^:*Ar*^*L/Y*^:*Gli1*^*CreER/+*^ mice. ***(C-D”)*** IHC analyses for Ki67 expression in different prostatic lobes of regenerated prostates from *R26*^*mTmG/+*^:*Gli1*^*CreER/+*^ or *R26*^*mTmG/+*^:*Ar*^*L/Y*^:*Gli1*^*CreER/+*^ mice. Scale bars, 20 μm.
(PDF)

S5 Fig Examination of gene expression using qRT-PCR. ***(A)*** Relative expression of probasin from Gli1-CreER driven GFP expressing cells and epithelial cells isolated from prostates of either *R26*^*mTmG/+*^:*Gli1*^*CreER/+*^ or *R26*^*mTmG/+*^:*Ar*^*L/Y*^:*Gli1*^*CreER/+*^ mice. Both Gli1CreER driven GFP expressing cells and prostatic epithelial cells were isolated and sorted by GFP or CD24 antibody, respectively. RNA samples were prepared and used to generate cDNA. The relative expression levels from three individual experiments were shown. ***(B—C)*** Fold changes in labeled expression of genes determined by qRT-PCR analysis using FACS-sorted GFP positive cells from either UGM tissues at day E16.5 (B) or prostate tissues at postnatal day 56 (C) isolated from *R26*^*mTmG/+*^:*Gli1*^*CreER/+*^ or *R26*^*mTmG/+*^:*Ar*^*L/Y*^:*Gli1*^*CreER/+*^ mice. Error bars indicate s.d.; *P < 0.05, ** *P* < 0.01; analyzed using 2-tailed students’ *t* test. (n = 3 replicates per data point).
(PDF)

S1 Table Quantification of AR and mGFP double positive cells per GFP positive cells of E18.5 UGS tissues. Supporting data for Fig 1M.
(PDF)

S2 Table Quantification of AR and mGFP double positive cells per GFP positive cells of P56 prostate tissues. Supporting data for Fig 4N right panel.
(PDF)

S3 Table Quantification of AR and mGFP double positive cells per GFP positive cells of different regenerated prostatic lobes. Supporting data for Fig 6M.
(PDF)

S4 Table Quantification of Ki67 and E-cadherin double positive cells per E-cadherin positive cells of different regenerated prostatic lobes. Supporting data for Fig 6N.
(PDF)

S5 Table Up-regulated and down-regulated gene list from Gli1-expressing cells from *R26^mTmG/+^:Ar^L/Y^:Gli1^CreER/+^* and *R26^mTmG/+^:Gli1^CreER/+^* mice. List of up-regulated and down-regulated genes from AR-deficient Gli1-expressing cells from *R26*^*mTmG/+*^:*Ar*^*L/Y*^:*Gli1*^*CreER/+*^ mice compared to normal Gli1-expressing cells from age- and sex-matched *R26*^*mTmG/+*^:*Gli1*^*CreER/+*^ controls. Supporting data for Fig 7C.
(PDF)

S6 Table Antibodies used for IHC and IF experiments in this study. (PDF)

S7 Table QRT-PCR primers used in this study. (PDF)
