# Supplementary material for: Loss of androgen signaling in mesenchymal sonic hedgehog responsive cells diminishes prostate development, growth, and regeneration
Source: PLoS Genet. 2020 Jan 13;16(1):e1008588. doi: 10.1371/journal.pgen.1008588 (PMC6980684; doi:10.1371/journal.pgen.1008588)
Supplement: S1 Fig — Representative images of squamous metaplasia (A) and squamous cysts (B) with prominent central keratinization from prostate tissues isolated from 8-week old R26mTmG/+:ArL/Y:Gli1CreER/+ mice. Scale bars, A, B, 200 μm; A’, B’, 20 μm. (PDF) [file pgen.1008588.s001.pdf]

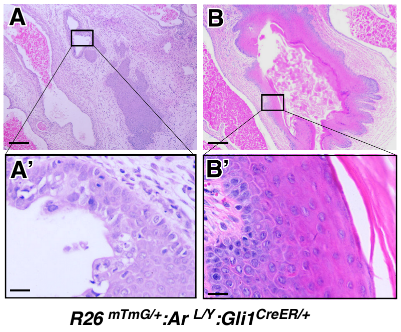

S1 Fig. Histopathologic analysis of postnatal prostates from mice with AR deletion in Gli1-expressing cells during embryogenesis. Representative images of squamous metaplasia ***(A)*** and squamous cysts ***(B)*** with prominent central keratinization from prostate tissues isolated from 8-week old $R26^{mTmG/+}$:$Ar^{L/Y}$:$Gli1^{CreER/+}$ mice. Scale bars, A and B, 200 µm; A' and B', 20 µm.
