# Supplementary material for: Loss of androgen signaling in mesenchymal sonic hedgehog responsive cells diminishes prostate development, growth, and regeneration
Source: PLoS Genet. 2020 Jan 13;16(1):e1008588. doi: 10.1371/journal.pgen.1008588 (PMC6980684; doi:10.1371/journal.pgen.1008588)
Supplement: S2 Fig — (A-B) Fluorescence micrographs of mT and mG expression from P56 prostate tissues from male mice of the indicated genotypes. Scale bars, A-B, 20 μm; A’-B’, 10 μm. (C1 to J5) Images of triple-immunofluorescent staining of prostatic tissues with the indicated antibodies. White arrows indicate AR and GFP double positive cells (C5). Pink arrows indicate triple positive cells (D5 and E5). Blue arrows indicate AR-negative, GFP-positive, and E-cadherin negative cells (G5). Green arrows indicate AR-negative, GFP-positive cells with either CD34 (H5) or SMA (I5) staining. Scale bars, C1-4, D1-4, E1-4, F1-4, G1-4, H1-4, I1-4, and J1-4, 20 μm; C5, D5, E5, F5, G5, H5, I5, and J5, 10 μm. (K) Quantification of AR and E-cadherin double positive cells per E-cadherin-positive cells (left panel) and AR and mGFP double positive cells per mGFP-positive cells (right panel) in P56 prostate tissues from male mice of the indicated genotypes. Error bars indicate s.d., analyzed using 2-tailed t-test; n = 6 mice per genotype. (PDF) [file pgen.1008588.s002.pdf]

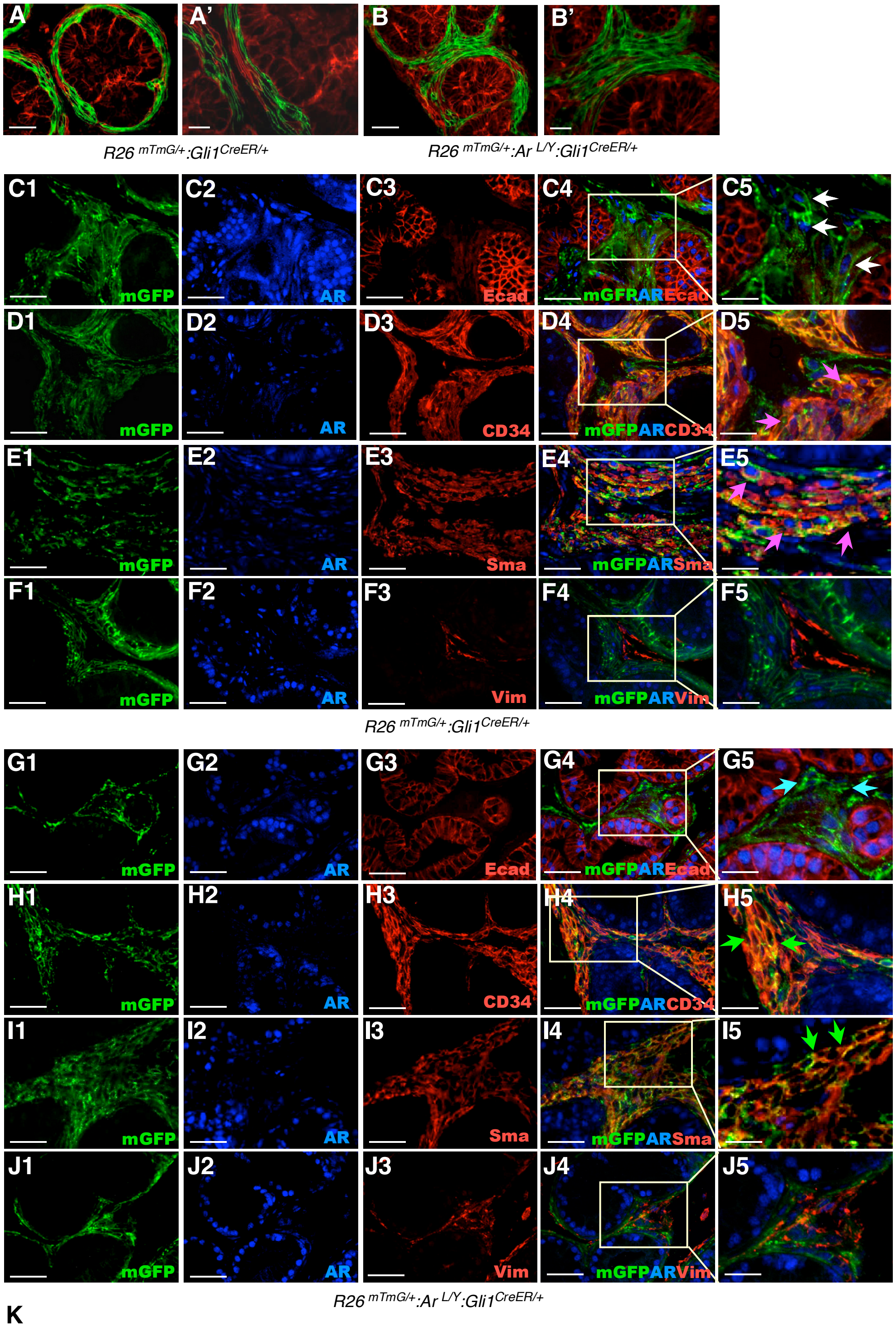

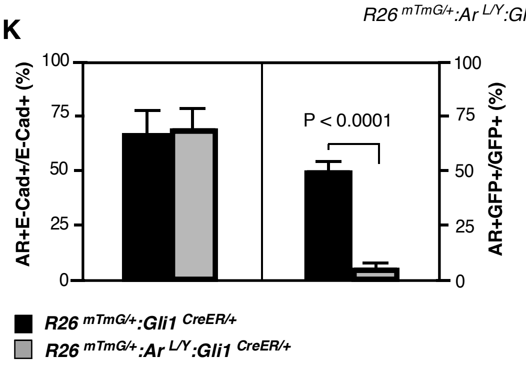

S2 Fig. Analyses of cellular properties of prostatic Gli1-expressing cells in mouse tissues. ***(A-B)*** Fluorescence micrographs of mT and mG expression from P56 prostate tissues from male mice of the indicated genotypes. Scale bars, A-B, 20 µm; A'-B', 10 µm. (***C1 to J5***) Images of triple-immunofluorescent staining of prostatic tissues with the indicated antibodies. White arrows indicate AR and GFP double positive cells (C5). Pink arrows indicate triple positive cells (D5 and E5). Blue arrows indicate AR-negative, GFP-positive, and E-cadherin negative cells (G5). Green arrows indicate AR-negative, GFP-positive cells with either CD34 (H5) or SMA (I5) staining. Scale bars, C1-4, D1-4, E1-4, F1-4, G1-4, H1-4, I1-4, and J1-4, 20 µm; C5, D5, E5, F5, G5, H5, I5, and J5, 10 µm. ***(K)*** Quantification of AR and E-cadherin double positive cells per E-cadherin-positive cells (left panel) and AR and mGFP double positive cells per mGFP-positive cells (right panel) in P56 prostate tissues from male mice of the indicated genotypes. Error bars indicate s.d., analyzed using 2-tailed t-test; n = 6 mice per genotype.
