# Supplementary material for: Loss of androgen signaling in mesenchymal sonic hedgehog responsive cells diminishes prostate development, growth, and regeneration
Source: PLoS Genet. 2020 Jan 13;16(1):e1008588. doi: 10.1371/journal.pgen.1008588 (PMC6980684; doi:10.1371/journal.pgen.1008588)
Supplement: S3 Fig — (A-F’) Representative H&E staining of prostatic lobes from P56 prostates isolated from R26mTmG/+:Gli1CreER/+ or R26mTmG/+:ArL/Y:Gli1CreER/+ mice. (G-L’) Representative H&E staining of prostatic lobes from P56 prostates isolated from androgen supplemented R26mTmG/+:Gli1CreER/+ and R26mTmG/+:ArL/Y:Gli1CreER/+ mice. (M-R’) Representative H&E staining of 8-week old implants from R26mTmG/+:Gli1CreER/+ or R26mTmG/+:ArL/Y:Gli1CreER/+ P14 prostatic lobes. Scale bars, A-R 100 μm; A’-R’ 20 μm. (PDF) [file pgen.1008588.s003.pdf]

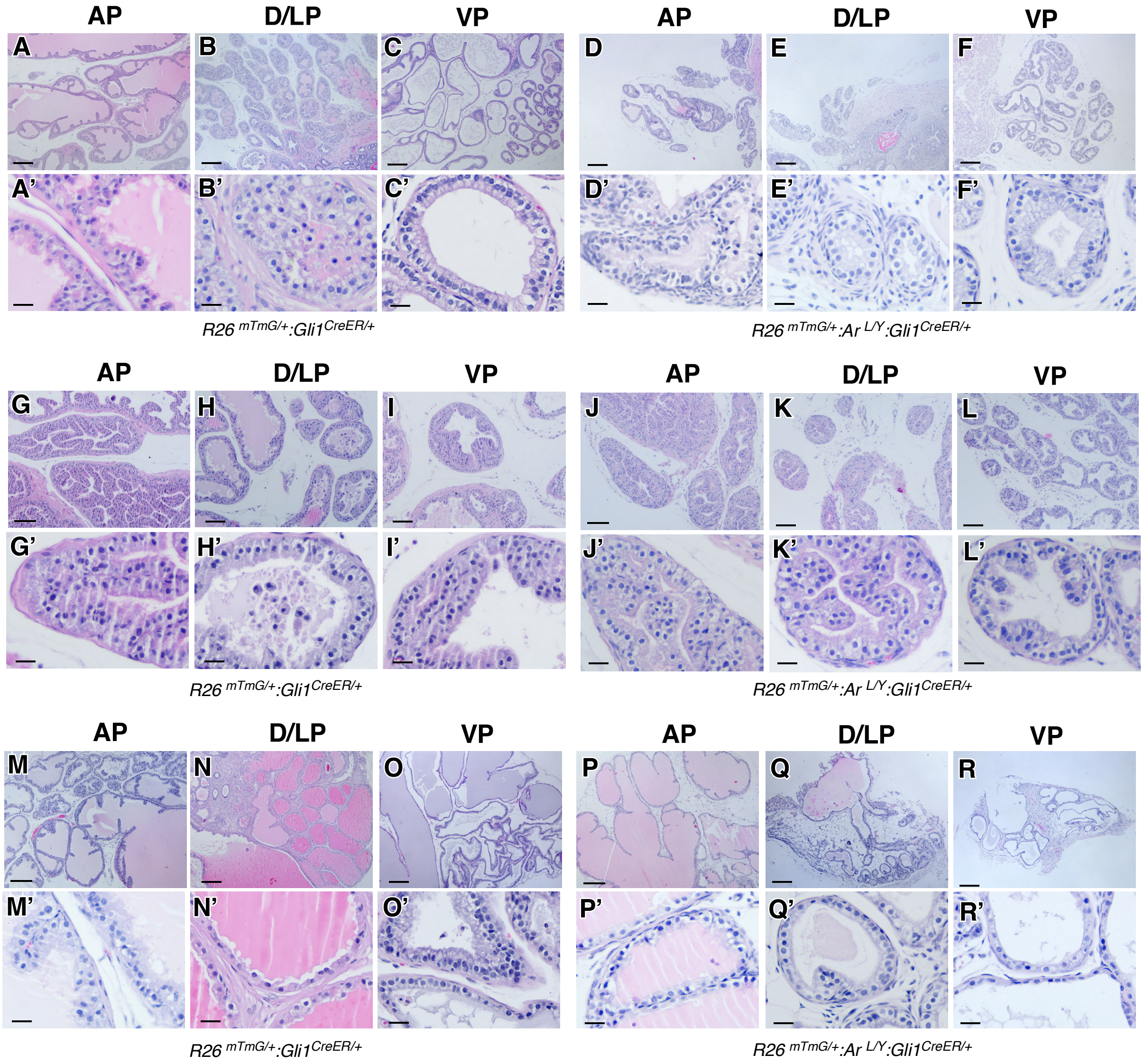

S3 Fig. Histologic analysis of prostatic lobes from *$R26^{mTmG/+}$:$Gli1^{CreER/+}$* or *$R26^{mTmG/+}$:$Ar^{L/Y}$:$Gli1^{CreER/+}$* mice. *(A-F')* Representative H&E staining of prostatic lobes from P56 prostates isolated from *$R26^{mTmG/+}$:$Gli1^{CreER/+}$* or *$R26^{mTmG/+}$:$Ar^{L/Y}$:$Gli1^{CreER/+}$* mice. ***(G-L')*** Representative H&E staining of prostatic lobes from P56 prostates isolated from androgen supplemented *$R26^{mTmG/+}$:$Gli1^{CreER/+}$* and *$R26^{mTmG/+}$:$Ar^{L/Y}$:$Gli1^{CreER/+}$* mice. ***(M-R')*** Representative H&E staining of 8-week old implants from *$R26^{mTmG/+}$:$Gli1^{CreER/+}$* or *$R26^{mTmG/+}$:$Ar^{L/Y}$:$Gli1^{CreER/+}$* P14 prostatic lobes. Scale bars, A-R 100 µm; A'-R' 20 µm.
