# Supplementary material for: Loss of androgen signaling in mesenchymal sonic hedgehog responsive cells diminishes prostate development, growth, and regeneration
Source: PLoS Genet. 2020 Jan 13;16(1):e1008588. doi: 10.1371/journal.pgen.1008588 (PMC6980684; doi:10.1371/journal.pgen.1008588)
Supplement: S4 Fig — (A-B”) IHC analyses for AR expression in different prostatic lobes of regenerated prostates from R26mTmG/+:Gli1CreER/+ or R26mTmG/+:ArL/Y:Gli1CreER/+ mice. (C-D”) IHC analyses for Ki67 expression in different prostatic lobes of regenerated prostates from R26mTmG/+:Gli1CreER/+ or R26mTmG/+:ArL/Y:Gli1CreER/+ mice. Scale bars, 20 μm. (PDF) [file pgen.1008588.s004.pdf]

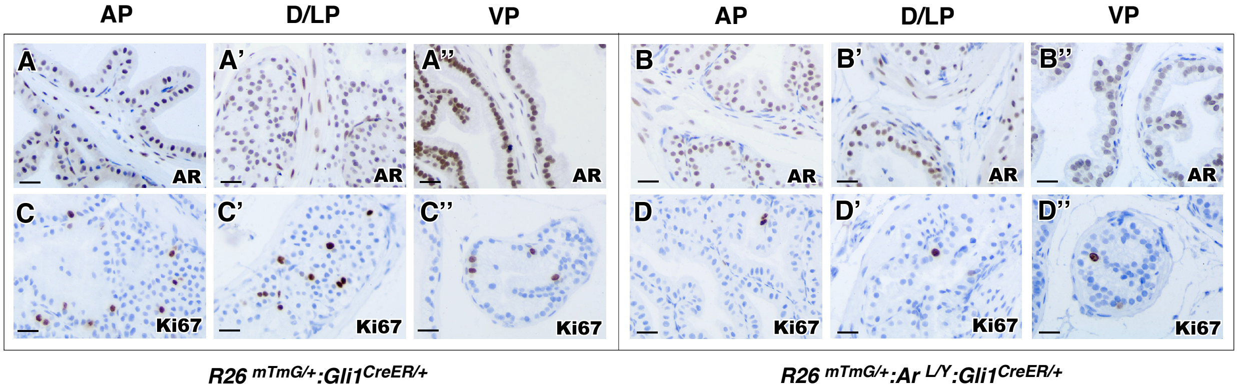

S4 Fig. Deletion of AR in Gli1-expressing cells after castration reduces their regenerative ability in adult prostates. ***(A-B'')*** IHC analyses for AR expression in different prostatic lobes of regenerated prostates from $R26^{mTmG/+}{:}Gli1^{CreER/+}$ or $R26^{mTmG/+}{:}Ar^{L/Y}{:}Gli1^{CreER/+}$ mice. ***(C-D'')*** IHC analyses for Ki67 expression in different prostatic lobes of regenerated prostate tissues from $R26^{mTmG/+}{:}Gli1^{CreER/+}$ or $R26^{mTmG/+}{:}Ar^{L/Y}{:}Gli1^{CreER/+}$ mice. Scale bars, 20 µm.
