# Supplementary material for: Loss of androgen signaling in mesenchymal sonic hedgehog responsive cells diminishes prostate development, growth, and regeneration
Source: PLoS Genet. 2020 Jan 13;16(1):e1008588. doi: 10.1371/journal.pgen.1008588 (PMC6980684; doi:10.1371/journal.pgen.1008588)
Supplement: S5 Fig — (A) Relative expression of probasin from Gli1-CreER driven GFP expressing cells and epithelial cells isolated from prostates of either R26mTmG/+:Gli1CreER/+ or R26mTmG/+:ArL/Y:Gli1CreER/+ mice. Both Gli1CreER driven GFP expressing cells and prostatic epithelial cells were isolated and sorted by GFP or CD24 antibody, respectively. RNA samples were prepared and used to generate cDNA. The relative expression levels from three individual experiments were shown. (B—C) Fold changes in labeled expression of genes determined by qRT-PCR analysis using FACS-sorted GFP positive cells from either UGM tissues at day E16.5 (B) or prostate tissues at postnatal day 56 (C) isolated from R26mTmG/+:Gli1CreER/+ or R26mTmG/+:ArL/Y:Gli1CreER/+ mice. Error bars indicate s.d.; *P < 0.05, ** P < 0.01; analyzed using 2-tailed students’ t test. (n = 3 replicates per data point). (PDF) [file pgen.1008588.s005.pdf]

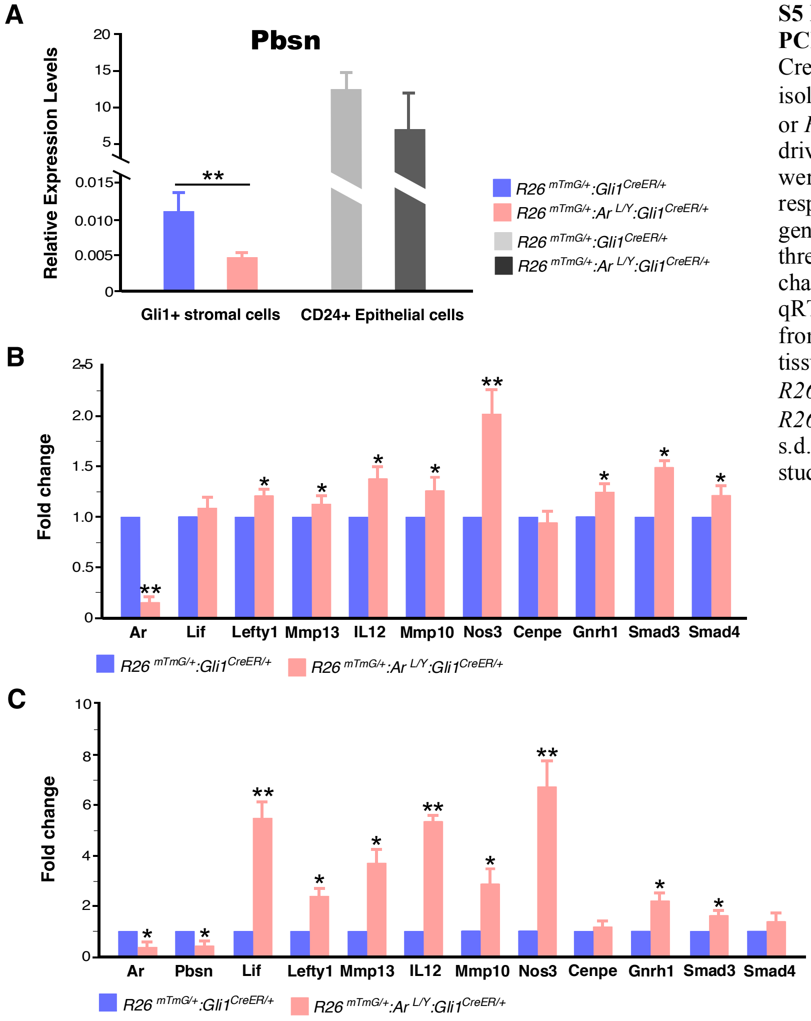

S5 Fig. Examination of gene expression using qRT-PCR. (*A*) Relative expression of probasin from Gli1-CreER driven GFP expressing cells and epithelial cells isolated from prostates of either $R26^{mTmG/+}:Gli1^{CreER/+}$ or $R26^{mTmG/+}:Ar^{L/Y}:Gli1^{CreER/+}$ mice. Both Gli1CreER driven GFP expressing cells and prostatic epithelial cells were isolated and sorted by GFP or CD24 antibody, respectively. RNA samples were prepared and used to generate cDNA. The relative expression levels from three individual experiments were shown. (*B - C*) Fold changes in labeled expression of genes determined by qRT-PCR analysis using FACS-sorted GFP positive cells from either UGM tissues at day E16.5 (*B*) or prostate tissues at postnatal day 56 (*C*) isolated from $R26^{mTmG/+}:Gli1^{CreER/+}$ and $Ar^{L/Y}:Gli1^{CreER/+}$ or $R26^{mTmG/+}:Ar^{L/Y}:Gli1^{CreER/+}$ mice. Error bars indicate s.d.; *$P < 0.05$, ** $P < 0.01$; analyzed using 2-tailed students' t test. ($n = 3$ replicates per data point).
