# Supplementary material for: Loss of androgen signaling in mesenchymal sonic hedgehog responsive cells diminishes prostate development, growth, and regeneration
Source: PLoS Genet. 2020 Jan 13;16(1):e1008588. doi: 10.1371/journal.pgen.1008588 (PMC6980684; doi:10.1371/journal.pgen.1008588)
Supplement: S1 Table — Supporting data for Fig 1M. (PDF) [file pgen.1008588.s006.pdf]

Table S1. Quantification of AR and mGFP double positive cells per GFP positive cells of E18.5 UGS tissues.

Supporting data for Figure 1M						
	$R26^{mTmGL/+}$:$Gli1^{CreER/+}$			$R26^{mTmGL/+}$:$Ar^{L/Y}$:$Gli1^{CreER/+}$		
	**GFP+AR+**	**Total GFP+**	**Percentage**	**GFP+AR+**	**Total GFP+**	**Percentage**
**#1**	141	143	98.6	25	138	18.1
**#2**	56	58	96.6	44	249	17.7
**#3**	80	85	94.1	52	215	24.2
**#4**	64	68	94.1	99	170	58.2
**#5**	97	102	95.1	71	163	43.6
**#6**	48	49	98.0	46	234	19.7
**#7**	88	92	95.7	25	202	12.4
**#8**	64	68	94.1	49	116	42.2
**#9**	31	32	96.9	41	185	22.2
**#10**	77	85	90.6			
**#11**	82	86	95.3			
**#12**	69	76	90.8			
		**Mean**	**95.0**		**Mean**	**27.0**
		**S.D.**	**2.4**		**S.D.**	**14.6**
