# Supplementary material for: Loss of androgen signaling in mesenchymal sonic hedgehog responsive cells diminishes prostate development, growth, and regeneration
Source: PLoS Genet. 2020 Jan 13;16(1):e1008588. doi: 10.1371/journal.pgen.1008588 (PMC6980684; doi:10.1371/journal.pgen.1008588)
Supplement: S2 Table — Supporting data for Fig 4N right panel. (PDF) [file pgen.1008588.s007.pdf]

Table S2. Quantification of AR and mGFP double positive cells per GFP positive cells of P56 prostate tissues.

Supporting data for Figure 4N right panel						
	**$R26^{mTmG/+}$:$Gli1^{CreER/+}$**			**$R26^{mTmG/+}$:$Ar^{L/Y}$:$Gli1^{CreER/+}$**		
	**GFP+AR+**	**Total GFP+**	**Percentage**	**GFP+AR+**	**Total GFP+**	**Percentage**
**#1**	21	48	43.8	3	60	5.0
**#2**	57	95	60.0	7	105	6.7
**#3**	33	56	58.9	3	127	2.4
**#4**	30	55	54.5	2	63	3.2
**#5**	39	67	58.2	3	70	4.3
**#6**	42	84	50.0	2	68	2.9
**#7**	32	56	57.1	0	70	0.0
**#8**	84	156	53.8	5	99	5.1
**#9**	46	104	44.2	2	53	3.8
**#10**	44	83	53.0	2	74	2.7
**#11**	76	138	55.1	3	77	3.9
**#12**	51	87	58.6	5	70	7.1
**#13**	23	44	52.3	3	56	5.4
**#14**	26	54	48.1			
		**Mean**	**53.6**		**Mean**	**4.0**
		**S.D.**	**5.1**		**S.D.**	**1.8**
