# Supplementary material for: Loss of androgen signaling in mesenchymal sonic hedgehog responsive cells diminishes prostate development, growth, and regeneration
Source: PLoS Genet. 2020 Jan 13;16(1):e1008588. doi: 10.1371/journal.pgen.1008588 (PMC6980684; doi:10.1371/journal.pgen.1008588)
Supplement: S3 Table — Supporting data for Fig 6M. (PDF) [file pgen.1008588.s008.pdf]

Table S3. Quantification of AR and mGFP double positive cells per GFP positive cells of different regenerated prostatic lobes.

Supporting data for figure 6M						
	**$R26^{mTmGL/+}$:$Gli1^{CreER/+}$**			**$R26^{mTmGL/+}$:$Ar^{L/Y}$:$Gli1^{CreER/+}$**		
**AP**	**GFP+AR+**	**Total GFP+**	**Percentage**	**GFP+AR+**	**Total GFP+**	**Percentage**
**#1**	5	41	12.2	9	39	23.1
**#2**	27	60	45.0	14	61	23.0
**#3**	14	45	31.1	17	42	40.5
**#4**	25	54	46.3	10	46	21.7
**#5**	19	44	43.2	3	24	12.5
**#6**	38	68	55.9	8	34	23.5
**#7**	24	59	40.7	5	34	14.7
**#8**	11	55	20.0	10	38	26.3
		**Mean**	**38.3**		**Mean**	**23.9**
		**S.D.**	**13.7**		**S.D.**	**7.9**

	**$R26^{mTmGL/+}$:$Gli1^{CreER/+}$**			**$R26^{mTmGL/+}$:$Ar^{L/Y}$:$Gli1^{CreER/+}$**		
**DLP**	**GFP+AR+**	**Total GFP+**	**Percentage**	**GFP+AR+**	**Total GFP+**	**Percentage**
**#1**	41	92	44.6	35	155	22.6
**#2**	58	134	43.3	9	61	14.8
**#3**	27	78	34.6	14	75	18.7
**#4**	11	61	18.0	1	61	1.6
**#5**	19	53	35.8	14	55	25.5
**#6**	29	51	56.9	15	65	23.1
**#7**	22	42	52.4	1	49	2.0
**#8**	32	71	45.1	4	27	14.8
		**Mean**	**41.1**		**Mean**	**17.0**
		**S.D.**	**11.2**		**S.D.**	**8.6**

	**$R26^{mTmGL/+}$:$Gli1^{CreER/+}$**			**$R26^{mTmGL/+}$:$Ar^{L/Y}$:$Gli1^{CreER/+}$**		
**VP**	**GFP+AR+**	**Total GFP+**	**Percentage**	**GFP+AR+**	**Total GFP+**	**Percentage**
**#1**	17	61	27.9	5	52	9.6
**#2**	16	30	53.3	4	51	7.8
**#3**	23	51	45.1	6	33	18.2
**#4**	31	77	40.3	3	44	6.8
**#5**	19	44	43.2	0	23	0.0
**#6**	15	39	38.5	4	46	8.6
**#7**	29	66	43.9	6	50	12
**#8**	15	53	28.3	2	38	5.3
		**Mean**	**39.2**		**Mean**	**8.6**
		**S.D.**	**8.0**		**S.D.**	**5.3**
