# Supplementary material for: Loss of androgen signaling in mesenchymal sonic hedgehog responsive cells diminishes prostate development, growth, and regeneration
Source: PLoS Genet. 2020 Jan 13;16(1):e1008588. doi: 10.1371/journal.pgen.1008588 (PMC6980684; doi:10.1371/journal.pgen.1008588)
Supplement: S4 Table — Supporting data for Fig 6N. (PDF) [file pgen.1008588.s009.pdf]

**Table S4. Quantification of Ki67 and E-cadherin double positive cells per E-cadherin positive cells of different regenerated prostatic lobes.**

| Supporting data for figure 6N | | | | | | |
|---|---|---|---|---|---|---|
| | **$R26^{mTmGL/+}$:$Gli1^{CreER/+}$** | | | **$R26^{mTmGL/+}$:$Ar^{L/Y}$:$Gli1^{CreER/+}$** | | |
| **AP** | **Ecad+Ki67+** | **Total Ecad+** | **Percentage** | **Ecad+Ki67+** | **Total Ecad+** | **Percentage** |
| **#1** | 16 | 561 | 2.9 | 3 | 166 | 1.8 |
| **#2** | 7 | 508 | 1.4 | 0 | 203 | 0.0 |
| **#3** | 14 | 189 | 7.4 | 1 | 209 | 0.5 |
| **#4** | 10 | 245 | 4.1 | 2 | 278 | 0.7 |
| **#5** | 9 | 417 | 2.2 | 3 | 369 | 0.8 |
| | | **Mean** | **2.9** | | **Mean** | **0.8** |
| | | **S.D.** | **2.1** | | **S.D.** | **0.7** |

| | **$R26^{mTmGL/+}$:$Gli1^{CreER/+}$** | | | **$R26^{mTmGL/+}$:$Ar^{L/Y}$:$Gli1^{CreER/+}$** | | |
|---|---|---|---|---|---|---|
| **DLP** | **Ecad+Ki67+** | **Total Ecad+** | **Percentage** | **Ecad+Ki67+** | **Total Ecad+** | **Percentage** |
| **#1** | 4 | 104 | 3.8 | 1 | 144 | 0.7 |
| **#2** | 9 | 241 | 3.7 | 2 | 210 | 1.0 |
| **#3** | 14 | 358 | 3.9 | 0 | 119 | 0.0 |
| **#4** | 18 | 403 | 4.5 | 1 | 400 | 0.3 |
| **#5** | 16 | 470 | 3.4 | 3 | 398 | 0.6 |
| | | **Mean** | **3.9** | | **Mean** | **0.5** |
| | | **S.D.** | **0.3** | | **S.D.** | **0.4** |

| | **$R26^{mTmGL/+}$:$Gli1^{CreER/+}$** | | | **$R26^{mTmGL/+}$:$Ar^{L/Y}$:$Gli1^{CreER/+}$** | | |
|---|---|---|---|---|---|---|
| **VP** | **Ecad+Ki67+** | **Total Ecad+** | **Percentage** | **Ecad+Ki67+** | **Total Ecad+** | **Percentage** |
| **#1** | 16 | 293 | 5.5 | 2 | 183 | 1.1 |
| **#2** | 4 | 349 | 1.1 | 1 | 186 | 0.5 |
| **#3** | 7 | 111 | 6.3 | 3 | 354 | 0.9 |
| **#4** | 9 | 320 | 2.8 | 2 | 312 | 0.6 |
| **#5** | 8 | 201 | 4.0 | 1 | 238 | 0.4 |
| | | **Mean** | **3.5** | | **Mean** | **0.7** |
| | | **S.D.** | **1.8** | | **S.D.** | **0.3** |
