# Supplementary material for: Loss of androgen signaling in mesenchymal sonic hedgehog responsive cells diminishes prostate development, growth, and regeneration
Source: PLoS Genet. 2020 Jan 13;16(1):e1008588. doi: 10.1371/journal.pgen.1008588 (PMC6980684; doi:10.1371/journal.pgen.1008588)
Supplement: S5 Table — List of up-regulated and down-regulated genes from AR-deficient Gli1-expressing cells from R26mTmG/+:ArL/Y:Gli1CreER/+ mice compared to normal Gli1-expressing cells from age- and sex-matched R26mTmG/+:Gli1CreER/+ controls. Supporting data for Fig 7C. (PDF) [file pgen.1008588.s010.pdf]

Table S5. Up-regulated and down-regulated gene list from Gli1-expressing cells from *R26^{mTmG/+}:Ar^{L/Y}:Gli1^{CreER/+}* and *R26^{mTmG/+}:Gli1^{CreER/+}* mice. List of up-regulated and down-regulated genes from AR-deficient Gli1-expressing cells from *R26^{mTmG/+}:Ar^{L/Y}:Gli1^{CreER/+}* mice compared to normal Gli1-expressing cells from age- and sex-matched *R26^{mTmG/+}:Gli1^{CreER/+}* controls. Supporting data for Figure 7C.

Symbol	logFC	PValue	FDR
Mrgprg	-6.94372	4.44E-14	2.35E-11
Gbp1	-6.06028	1.31E-08	2.79E-06
Impg1	-5.85428	1.01E-07	1.86E-05
Pdcd1	-5.61733	8.43E-07	0.000115
Tmed6	-5.35973	1.34E-05	0.001187
Lyz1	-5.20423	3.08E-25	5.01E-22
Dsg4	-5.19518	4.2E-05	0.003236
Fpr2	-5.18795	4.2E-05	0.003236
Ugt2b1	-4.85231	0.000244	0.013311
Fer1l4	-4.84369	0.000244	0.013311
Nsun4	-4.83052	0.000444	0.021161
Ceacam2	-4.82529	0.000444	0.021161
Cyp2ab1	-4.8192	0.000444	0.021161
Tsix	-4.75041	1.07E-08	2.35E-06
Tmprss11f	-4.69864	0.000812	0.034001
Clca3	-4.49111	6.79E-13	3.12E-10
Slco4c1	-4.48317	0.001498	0.053181
Pcdh15	-4.44923	3.48E-10	9.41E-08
Muc20	-4.37178	1.18E-21	1.31E-18
Cd209d	-4.35875	1.04E-12	4.68E-10
Ces1e	-4.11161	3.33E-10	9.13E-08
Slc10a5	-4.04895	8.5E-08	1.6E-05
Ccl22	-3.87422	1.48E-14	8.24E-12
Cd209b	-3.77348	4.38E-19	3.96E-16
Slc25a18	-3.75668	0.000123	0.007862
Clec2i	-3.75591	1.99E-06	0.000243
Klk1b22	-3.71922	2.71E-13	1.36E-10
Il5	-3.6791	0.000208	0.012013
Retnla	-3.67847	2.82E-34	9.93E-31
Fmo3	-3.62323	4.5E-19	3.96E-16
Agr2	-3.55283	1.14E-19	1.09E-16
Atoh1	-3.51985	8.64E-10	2.15E-07
Cyp2b10	-3.51421	8.64E-10	2.15E-07
Sox2ot	-3.50295	0.001032	0.040011
Nkx2-1	-3.42914	0.001032	0.040011

Fcna	-3.42295	1.23E-27	2.17E-24
Lgals12	-3.41357	0.001032	0.040011
Rnf183	-3.40456	0.001032	0.040011
Dkkl1	-3.40141	4.87E-19	4.12E-16
Cd200r2	-3.39204	5.33E-05	0.003914
Lhx5	-3.36123	3.47E-08	6.93E-06
Folr1	-3.32528	3.21E-20	3.39E-17
Pbsn	-3.2369	0.00014	0.008773
Oca2	-3.23346	0.000226	0.012683
Lrrc31	-3.2025	1.4E-09	3.4E-07
C6	-3.18096	2.06E-12	8.73E-10
Ccl6	-3.14635	4.11E-25	6.21E-22
Olfm4	-3.12451	7.01E-24	9.26E-21
Csmd3	-3.00171	3.25E-06	0.000379
Ccl8	-2.98696	4.92E-24	6.92E-21
Trim67	-2.95176	0.000976	0.038757
Slc5a8	-2.8842	2.12E-12	8.79E-10
Tspan32	-2.88148	1.04E-05	0.000977
Cxcl17	-2.87343	1.17E-14	6.67E-12
Gria2	-2.79498	0.000126	0.008055
Scn4b	-2.77208	3.6E-05	0.002827
Nat8l	-2.76775	2.29E-05	0.00192
Slc26a9	-2.75685	3.4E-05	0.002701
Ugt8a	-2.74798	0.000834	0.034479
Entpd8	-2.73648	3.92E-11	1.31E-08
Ifitm6	-2.70765	0.000192	0.011413
Tmprss4	-2.70429	0.000192	0.011413
Pglyrp1	-2.69502	5.09E-12	1.99E-09
Ccdc89	-2.68886	0.001311	0.048424
Cd300ld	-2.64965	6.65E-14	3.43E-11
Pydc4	-2.64836	3.35E-06	0.000382
Tnfrsf14	-2.62166	3.59E-05	0.002827
Oit1	-2.61983	2.04E-17	1.54E-14
Spink5	-2.56461	3.35E-06	0.000382
Eci3	-2.52742	4.64E-07	7.01E-05
Wfdc15b	-2.47791	5.37E-10	1.4E-07
Ctcfl	-2.46487	4.59E-06	0.000484
Ms4a8a	-2.42837	1.61E-05	0.001398
Pla2g4e	-2.4274	0.000549	0.025177
Dhrs9	-2.41424	6.46E-12	2.42E-09
Psca	-2.40773	5.68E-13	2.73E-10
Amd2	-2.4069	0.000549	0.025177
Ccl9	-2.35383	3.05E-14	1.65E-11

Car2	-2.33696	1.99E-16	1.4E-13
Hal	-2.29917	3.41E-09	8.01E-07
Tlr8	-2.2886	3.97E-08	7.83E-06
Tmem8	-2.2877	1.07E-10	3.2E-08
Oas1g	-2.26196	1.61E-05	0.001398
Oas1c	-2.25077	1.76E-06	0.000223
Lrrc26	-2.23969	1.08E-12	4.75E-10
Galnt9	-2.23199	5.02E-05	0.003722
Rit2	-2.20082	4.03E-07	6.39E-05
Ugt1a1	-2.18176	2.15E-06	0.000259
Lrrtm4	-2.17837	7.08E-05	0.004955
Cxcr3	-2.16713	6.03E-05	0.004318
Cd200r1	-2.14573	6.94E-06	0.000689
Ugt2b34	-2.13384	6.53E-12	2.42E-09
Folr2	-2.10596	5.11E-13	2.51E-10
Mpz	-1.91599	1.4E-05	0.001231
Lyve1	-1.89686	1.8E-10	5.21E-08
Tlr1	-1.85351	3.09E-07	5.03E-05
Ckm	-1.85038	0.000238	0.013179
Cd69	-1.84556	0.00111	0.042498
Rsad2	-1.7956	4.33E-10	1.16E-07
Syt4	-1.75835	1.16E-06	0.000153
Ar	-1.75036	2.35E-10	6.53E-08
Lst1	-1.65266	0.001004	0.039488
Alox5ap	-1.6442	7.33E-07	0.000103
Cd84	-1.64392	1.48E-06	0.000191
Rorc	-1.59355	3.02E-05	0.002456
Dok2	-1.57665	1E-06	0.000133
H2-DMb2	-1.57623	0.000131	0.008313
Slc5a1	-1.54665	0.000429	0.021103
Alox5	-1.54124	5.05E-06	0.000516
Cd4	-1.51387	8.37E-06	0.000807
Srp54a	-1.51233	0.000346	0.017696
Fcer1g	-1.50873	1.1E-07	2.01E-05
Myo5c	-1.48147	3.09E-05	0.002491
Ap1m2	-1.45932	1.09E-05	0.001002
Siglece	-1.45139	5.6E-05	0.004081
Dnahc7b	1.458218	0.000663	0.029017
Slc22a1	1.46127	0.000222	0.012598
Nebl	1.483424	2.38E-06	0.000282
Eps8l1	1.491165	2.18E-06	0.00026
Atp6v0d2	1.501546	0.000435	0.021161
Gm9008	1.514597	0.000821	0.034078

Otop1	1.515448	0.001034	0.040013
Lif	1.518707	4.52E-08	8.76E-06
Nos3	1.521591	0.000816	0.034001
Pif1	1.533107	0.000296	0.015532
Rnaset2b	1.539037	0.000122	0.007862
Rasgef1a	1.542688	0.000435	0.021161
Igsf9b	1.547291	6.25E-05	0.004447
Slc5a7	1.554231	0.000661	0.028986
Nav3	1.565037	8.62E-08	1.61E-05
Gm13251	1.574247	0.000256	0.013778
Tmem144	1.576141	2.27E-07	3.87E-05
Gm11722	1.577421	6.73E-05	0.004723
Slc39a4	1.583095	3.56E-06	0.000396
Kank4	1.587279	3.42E-07	5.52E-05
Rin1	1.600372	4.05E-07	6.39E-05
Mmp10	1.602304	4.2E-07	6.52E-05
Aicda	1.603042	0.000816	0.034001
Xkr4	1.616554	5.24E-05	0.003861
Klk1	1.627055	4.1E-07	6.42E-05
Cdh23	1.630752	0.000643	0.028438
Rgs20	1.631421	0.001319	0.048469
Il2rb	1.646975	6.38E-05	0.004494
Sst	1.647198	0.000507	0.02365
Nos1	1.649362	0.000742	0.031878
Tmem169	1.654751	0.000612	0.027199
Cenpe	1.661998	9.15E-07	0.000123
Ivl	1.670578	3.34E-05	0.002674
Gm13219	1.684811	2.35E-05	0.001958
Tm4sf4	1.686795	3.94E-06	0.000426
Gm17414	1.694018	0.000293	0.015483
A530054K11Rik	1.700378	8.32E-07	0.000114
Tns4	1.709034	1.38E-07	2.47E-05
Lefty1	1.710308	0.000486	0.022712
Tuba8	1.721447	6.57E-06	0.000658
Ttyh2	1.747712	4.96E-10	1.31E-07
Epm2a	1.75874	7.49E-08	1.43E-05
Gsg2	1.769154	0.000217	0.012346
Dgkk	1.779561	0.00126	0.047138
RP24-201C14.6	1.800582	0.000205	0.011956
Rnase1	1.82974	1.39E-09	3.4E-07
Stag3	1.8412	1.92E-06	0.000237
Gldn	1.844976	1.5E-06	0.000192
Otud7a	1.849187	0.00111	0.042498

D130062J21Rik	1.879199	0.000608	0.02711
Spred3	1.895203	3.38E-05	0.002696
Igsf21	1.925181	1.4E-07	2.48E-05
Tmprss11a	1.943336	1.33E-08	2.8E-06
Ace2	1.949425	0.000373	0.018734
Hmgn2	2.036678	4.97E-12	1.98E-09
Olfr1033	2.039568	4.61E-06	0.000484
Abca4	2.072143	1.03E-05	0.000963
Sez6l	2.16921	7.47E-07	0.000104
Sycp1	2.174962	2.23E-10	6.28E-08
Epgn	2.245969	3.67E-06	0.000402
Prr9	2.309296	1.17E-14	6.67E-12
Sva	2.411262	5.44E-11	1.8E-08
Dsc1	2.412794	0.001218	0.046057
Sorcs1	2.514318	4.05E-05	0.003144
Olfr550	2.520046	0.000192	0.011413
Chga	2.610128	0.000531	0.024532
Fbp1	2.625462	1.71E-08	3.58E-06
Slpi	2.647711	0.000248	0.013479
Vip	2.698287	4.76E-06	0.000496
Ankrd45	2.711927	0.000683	0.029753
Pgbd1	2.775884	0.000447	0.021199
Kcnj2	2.939146	4.52E-06	0.00048
Bpifc	3.035769	0.000226	0.012683
Krt33a	3.063704	1.33E-06	0.000174
Il12b	3.07973	1.23E-15	7.65E-13
A930017K11Rik	3.341201	0.000603	0.027011
Marco	3.41895	1.74E-08	3.61E-06
AV064505	3.424061	0.000354	0.01797
Gm8113	3.48746	1.33E-07	2.4E-05
Gpr110	3.552125	0.000208	0.012013
Slc17a8	3.601123	1.26E-06	0.000165
Cnga2	3.618358	0.000123	0.007862
Slc14a1	3.674538	2.56E-30	5.41E-27
Btla	3.77214	4.32E-05	0.003302
Chi3l3	3.795815	2.06E-07	3.61E-05
A530099J19Rik	3.958872	9.25E-06	0.00088
Slc30a3	3.961321	9.25E-06	0.00088
Svs5	3.964478	1.47E-32	3.89E-29
Hba-a1	4.042177	7.34E-11	2.29E-08
Krt34	4.453912	0.001498	0.053181
Alox12e	4.516058	9.97E-12	3.63E-09
Hbb-b2	4.570024	0.001498	0.053181

Hba-a2	4.572032	0.000812	0.034001
Dsg3	4.575689	0.000812	0.034001
Zfp804b	4.764199	0.000444	0.021161
Ccnb1ip1	4.777473	0.000444	0.021161
Gnrh1	4.778367	0.000444	0.021161
Sprr2f	4.957186	8.32E-11	2.55E-08
Caln1	5.280418	1.34E-05	0.001187
Sprr2g	5.285713	1.34E-05	0.001187
Gpr82	5.427216	4.35E-06	0.000469
Mmp7	7.142512	6.1E-16	4.03E-13
Hbb-b1	7.403376	5.59E-18	4.37E-15
