# Supplementary material for: Loss of androgen signaling in mesenchymal sonic hedgehog responsive cells diminishes prostate development, growth, and regeneration
Source: PLoS Genet. 2020 Jan 13;16(1):e1008588. doi: 10.1371/journal.pgen.1008588 (PMC6980684; doi:10.1371/journal.pgen.1008588)
Supplement: S6 Table — (PDF) [file pgen.1008588.s011.pdf]

Table S6. Antibodies used for IHC and IF experiments in this study.

Antibody	Vendors and Cat #	Species	Working dilution
GFP	Cell Signaling #2956	rabbit IgG	1:200
GFP	Cell Signaling #2955	mouse IgG	1:200
GFP	Abcam #ab13970	chicken IgG	1:2000
AR	ThermoFisher #PA5-16750	rabbit IgG	1:500
E-cadherin	BD Transduction Laboratories #610182	mouse IgG	1:200
SMA	Sigma #A5228	mouse IgG	1:4000
Vimentin	BioLegend #919101	chicken IgG	1:2000
CD34	Invitrogen #14-0341-82	rat IgG	1:50
Ki67	Cell Signaling #9129	rabbit IgG	1:500
T□RII	Santa Cruz #sc-220	rabbit IgG	1:200
Biotinylated anti-rabbit	Vector Laboratories #BA-1000	goat IgG	1:750
Biotinylated anti-rabbit	Vector Laboratories #BA-2000	goat IgG	1:750
Goat anti-rabbit 488	Invitrogen #A11034	goat IgG	1:500
Goat anti-mouse 488	Invitrogen #A11001	goat IgG	1:500
Goat anti-rabbit 594	Invitrogen #A11012	goat IgG	1:500
Goat anti-mouse 594	Invitrogen #A11005	goat IgG	1:500
Goat anti-chicken 647	Invitrogen #A31571	goat IgG	1:200
