# Supplementary material for: Loss of androgen signaling in mesenchymal sonic hedgehog responsive cells diminishes prostate development, growth, and regeneration
Source: PLoS Genet. 2020 Jan 13;16(1):e1008588. doi: 10.1371/journal.pgen.1008588 (PMC6980684; doi:10.1371/journal.pgen.1008588)
Supplement: S7 Table — (PDF) [file pgen.1008588.s012.pdf]

Table S7. qRT-PCR primers used in this study.

Genes	Primer name	Sequences	Amplicon (bp)
AR	qPCR-AR-F	5'- AAA CTT CTT TCG CTG GGG CTT C - 3'	90
	qPCR-AR-R	5'- AGA ACA GAA CAC TAG CGC TTG G - 3'	
Probasin	qPCR-PBSN-F	5'- CAC AGT ATG AAG GGA GCA TAG - 3'	105
	qPCR-PBSN-R	5'- CCA CTC GTG TGA CAT CAT T - 3'	
LIF	qPCR-LIF-F	5'- ACG GCA ACC TCA TGA AC - 3'	92
	qPCR-LIF-R	5'- CCC TTG AGC TGT GTA ATA GG - 3'	
LEFTY1	qPCR-LEFTY1-F	5'- CAC ACA AGT TGG TTC GTT TC - 3'	111
	qPCR-LEFTY1-R	5'- TGC CTT GAG CTC CAT AGT - 3'	
MMP13	qPCR-MMP13-F	5'- GCA GTT CCA AAG GCT ACA A - 3'	77
	qPCR-MMP13-R	5'- CTT GGA GTG ATC CAG ACC TA - 3'	
IL12	qPCR-IL12-F	5'- GTC CAG GCA CAT CAG ACC - 3'	100
	qPCR-IL12-R	5'- GAG TGG AGA CAC CAG CCA A - 3'	
MMP10	qPCR-MMP10-F	5'- GGG TCT CTT TCA TTC CGA CAA G - 3'	93
	qPCR-MMP10-R	5'- TCA TCT TGA GAA AGG TGG AAG TTA G - 3'	
NOS3	qPCR-NOS3-F	5'- ATC CTA ACT TGC CCT GCA TC - 3'	235
	qPCR-NOS3-R	5'- CAG GTT GTA GCC CTT TGA TCT - 3'	
CENPE	qPCR-CENPE-F	5'- AGC ATT GGG CTC GTG AAT AA - 3'	121
	qPCR-CENPE-R	5'- GTG ACT TCA GGT ACT GAT CCA AG - 3'	
GNRH1	qPCR-GNRH1-F	5'- TCT ACT GCT GAC TGT GTG TTT G - 3'	135
	qPCR-GNRH1-R	5'- GCC ATT TGA TCC ACC TCC TT - 3'	
SMAD3	qPCR-SMAD3-F	5'- CAC GCA GAA CGT GAA CAC C - 3'	101
	qPCR-SMAD3-R	5'- GGC AGT AGA TAA CGT GAG GGA - 3'	
SMAD4	qPCR-SMAD4-F	5'- ATG TGA CAG TGT CTG TGT GAA - 3'	82
	qPCR-SMAD4-R	5'- CTG CAG TGT TAA TCC TGA GAG A - 3'	
PPIA	qPCR-PP1A-F	5'- TGT GCC AGG GTG GTG ACT TT - 3'	144
	qPCR-PP1A-R	5'- CGT TTG TGT TTG GTC CAG CAT - 3'	
